# A sequentially targeted and pathology-responsive nanoplatform for synergistic treatment of dry eye disease via concurrent anti-inflammation and mitochondrial ROS scavenging

**DOI:** 10.1186/s12951-026-04365-7

**Published:** 2026-05-22

**Authors:** Liandi Huang, Xin Liu, Ying Yuan, Bilian Ke

**Affiliations:** 1 Department of Ophthalmology, Shanghai General Hospital, Shanghai Jiao Tong University School of Medicine, No. 100 Haining Road, Shanghai, 200080 China; 2 National Clinical Research Center for Eye Diseases, Shanghai Engineering Center for Visual Science and Photomedicine, Shanghai Engineering Center for Precise Diagnosis and Treatment of Eye Diseases, Shanghai Key Laboratory of Ocular Fundus Diseases, Shanghai, People’s Republic of China; 3 Department of Ophthalmology, Ren Ji Hospital, Shanghai Jiao Tong University School of Medicine, No. 160 Pujian Road, Shanghai, 200127 China

**Keywords:** Sequential targeting, Dry eye disease, CCL2, Mitochondrial targeting, Mesoporous polydopamine, Reactive oxygen species, Pathology-responsive delivery, Nanoscavenger

## Abstract

The treatment of complex inflammatory diseases like dry eye disease (DED) is hampered by the intertwined pathologies of inflammation and oxidative stress, which form a self-amplifying vicious cycle. Here, we report an intelligent nanoplatform (CFMPDA) designed to actively navigate and disrupt this cycle through sequential, pathology-responsive targeting. CFMPDA is constructed by encapsulating the anti-inflammatory agent Fuziline within a mesoporous polydopamine (MPDA) nanoscavenger and conjugating CCL2-targeting antibodies on its surface. This design enables a two-stage therapeutic strategy: first, CCL2-mediated anchoring to the inflamed cornea, providing disease severity-dependent retention that overcomes rapid ocular clearance; second, subsequent mitochondrial targeting to deliver combined anti-inflammatory and antioxidant therapy directly to the organelle generating pathogenic ROS. In vivo, CFMPDA demonstrated potent, synergistic efficacy in a DED mouse model, simultaneously suppressing the inflammatory cascade (IL-1β, IL-6, TNF-α, MMP9, T cell infiltration) and quenching oxidative stress, leading to comprehensive tissue repair and functional recovery that surpassed the clinical standard-Fluorometholone. This work establishes a versatile nanotechnology blueprint—responsive bio-interfacing coupled with programmed subcellular delivery-for the synergistic treatment of inflammatory disorders characterized by chemokine upregulation and mitochondrial dysfunction.

**Graphical Abstract:**

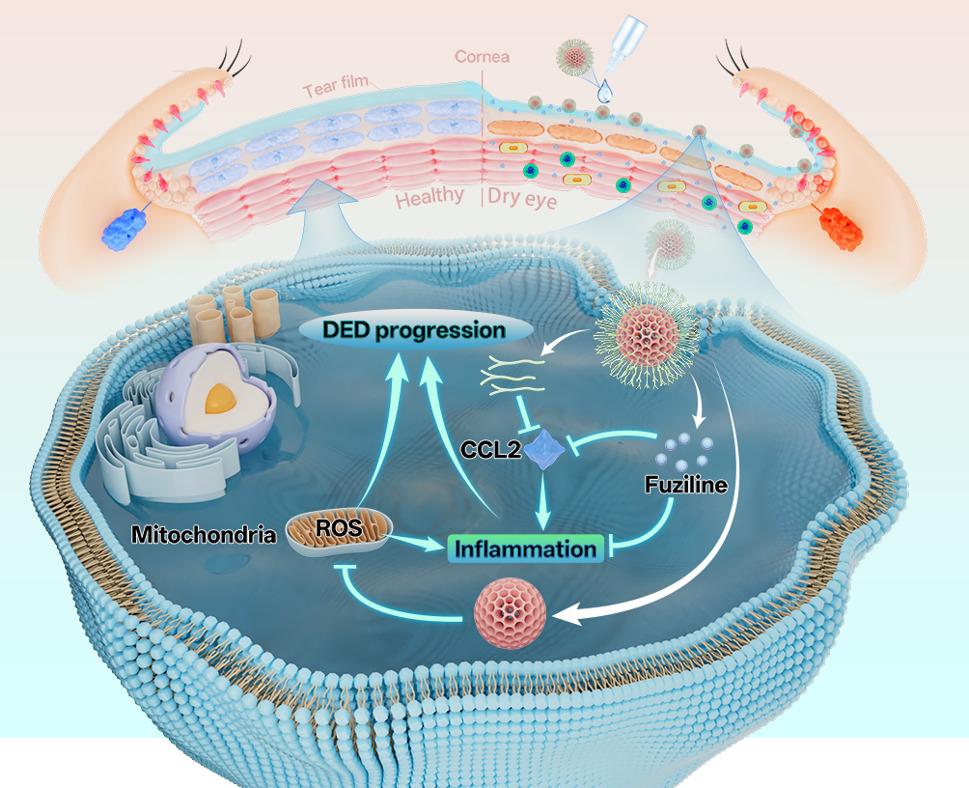

**Supplementary Information:**

The online version contains supplementary material available at 10.1186/s12951-026-04365-7.

## Introduction

Dry eye disease (DED) is a prevalent and debilitating ocular disorder, driven by a self-perpetuating vicious cycle wherein inflammatory signaling and oxidative stress reciprocally amplify across tissue, cellular, and subcellular hierarchies [1–3]. This pathological complexity poses a formidable therapeutic challenge. Conventional topical pharmacotherapy is fundamentally limited by rapid ocular clearance and poor corneal permeability, culminating in low drug bioavailability [4]. More critically, monotherapies fail to concurrently dismantle the intertwined pathological axes of inflammation and oxidation [5], underscoring an urgent need for innovative strategies that deliver coordinated multi-mechanistic intervention with spatial precision.

Nanotechnology offers a transformative platform to engineer advanced delivery systems [6]. However, to effectively disrupt resilient pathological circuits like those in DED [7], nanocarrier design must evolve from passive vehicles into actively navigating "smart" materials. The ideal system should embody a biomimetic, hierarchical logic: first, recognizing and anchoring to a disease-specific marker at the tissue interface to overcome biodistribution barriers; second, trafficking to specific intracellular pathological hubs to execute precise, localized therapeutic functions.

Guided by this principle, we deconstructed the pathogenic DED cycle to identify two prime therapeutic targets. At the tissue level, we identified the chemokine CCL2 as a master regulator of the inflammatory cascade and an ideal targeting epitope. In DED patients, CCL2 is markedly upregulated in tears, with its levels correlating positively with disease severity [8]. Its pathogenic role is defined by its capacity to orchestrate a complex pro-inflammatory network—driving immune cell recruitment, stimulating cytokine production (e.g., IL-1β, IL-6, TNF-α), and activating MMP9 [9–11]. At the subcellular level, mitochondrial dysfunction serves as a critical nexus, where excessive reactive oxygen species (ROS) generation fuels inflammation and propagates cellular damage [12]. We therefore hypothesized that a nanoplatform engineered for sequential targeting—first to CCL2 at the disease site, then to mitochondria within afflicted cells—could actively navigate the pathological landscape and deliver a concerted attack on the core drivers of the DED cycle.

Herein, we report the rational design, fabrication, and validation of CFMPDA, a hierarchical nanotherapeutic material that embodies this "sense-and-treat" strategy. The platform is architectured around a mesoporous polydopamine (MPDA) core, selected not merely as an inert carrier, but for its intrinsic dual bioactivity: serving as a potent nanoscale mitochondrial-site ROS scavenger and a high-capacity drug reservoir [13–15]. We further engineered this bioactive core by loading it with Fuziline—a natural diterpenoid alkaloid that inhibits the NLRP3/IL-1β axis [16–18]—and precisely conjugating its surface with CCL2-targeting antibodies to confer active, pathology-dependent recognition.

**Fig. 1:**
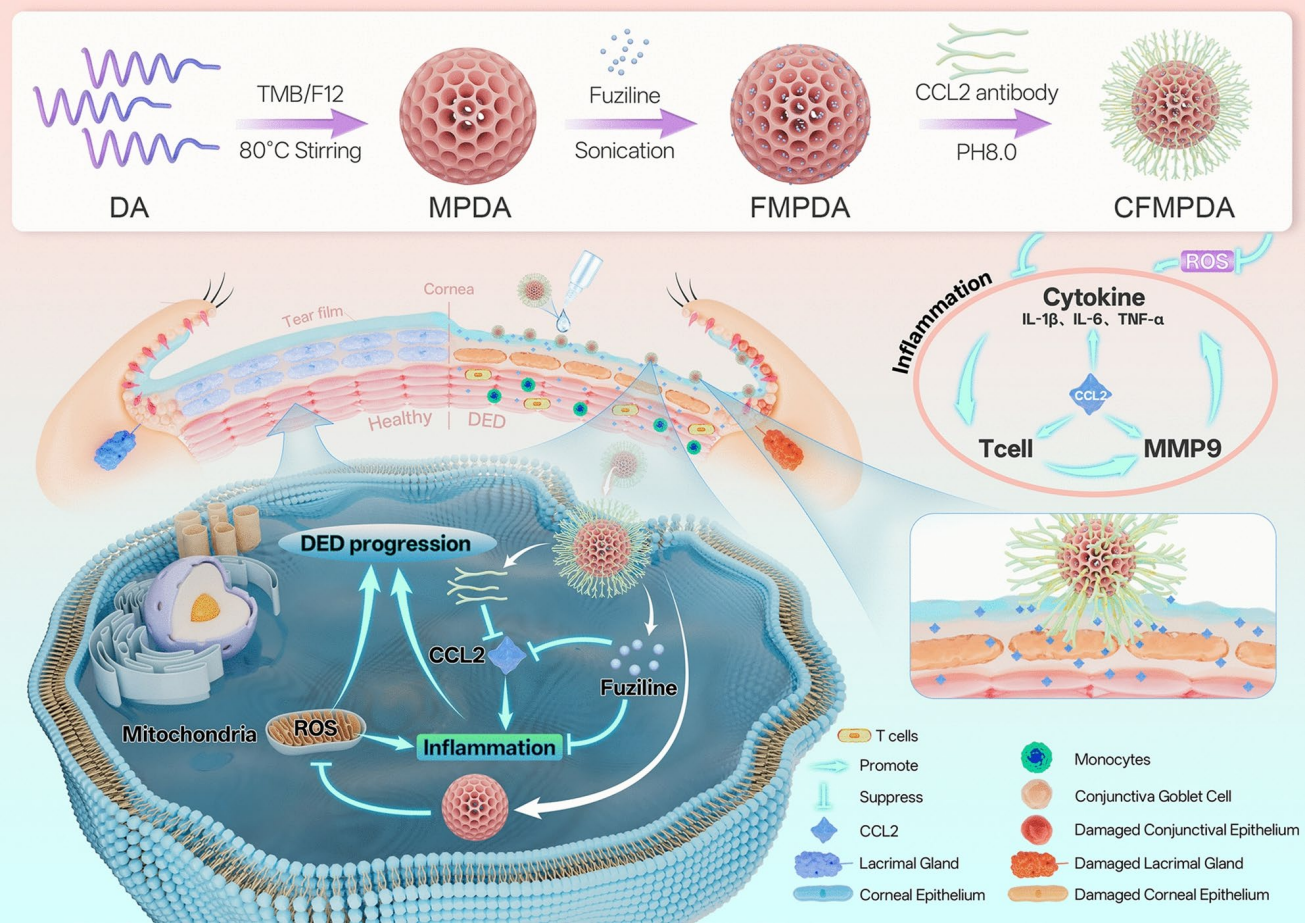
Schematic illustration of the preparation of CFMPDA and its therapeutic mechanism on DED

We demonstrate that this engineered material successfully executes its programmed therapeutic itinerary. It achieves: (1) CCL2-mediated, pathology-dependent anchoring and prolonged retention on the inflamed ocular surface; (2) efficient cellular internalization followed by precise mitochondrial localization; and (3) spatiotemporally coordinated action, releasing Fuziline to suppress the inflammatory cascade while utilizing the MPDA core to scavenge ROS at the mitochondrial source (Fig. 1). This orchestrated, materials-based intervention resulted in comprehensive therapeutic efficacy in a murine DED model, surpassing the clinical standard of care.

Beyond presenting a potent DED therapeutic, this work establishes a materials-centric blueprint for managing complex inflammatory diseases. By demonstrating how modular bioactivities—targeting, scavenging, and drug release—can be integrated into a single nanoscale entity through rational design, we provide a framework for creating intelligent systems capable of actively navigating biological hierarchies to achieve synergistic therapy.

## Materials and methods

### Materials

3,3',5,5'-Tetramethylbenzidine (TMB), F-127, Fuziline, and dopamine hydrochloride were purchased from Shanghai Macklin Biochemical Technology Co., Ltd. (Shanghai, China). Cy5 NHS Ester (CY5-SE), 2,2-diphenyl-1-picrylhydrazyl (DPPH), benzalkonium chloride (BAC), MitoSOX Red, adenosine 5'-triphosphate (ATP), xanthine oxidase (XOD), and lipopolysaccharides (LPS) were purchased from MedChemExpress (Monmouth Junction, NJ, USA). DAPI, Annexin V-FITC Apoptosis Detection Kit, Viability/Cytotoxicity Assay Kit, and Reactive Oxygen Species Assay Kit with CM-H2DCFDA were purchased from Beyotime (Nanjing, China). CCL2 antibody was purchased from Thermo Fisher Scientific (Waltham, MA, USA). Fluorometholone was purchased from Allergan Pharmaceuticals Ireland (Dublin, Ireland).

### Preparation of engineered nanoplatform

0.36 g TMB and 0.36 g F-127 were dissolved in a mixture of ethanol and water (25 ml ethanol, 25 ml water) with magnetic stirring at 400 rpm overnight. The solution was then heated to 80 °C, followed by the addition of polydopamine (90 mg) and ammonia (2 mL). After stirring at 400 rpm for 2 h, the mixture was transferred into a dialysis bag and dialyzed against double-distilled water. The dialysis water was changed at 2, 4, 8, 12, 24, and 36 h. The dialyzed solution was subsequently centrifuged at 40,000 rpm for 2.5 h. The collected sediment was washed twice with an acetone–ethanol mixed solvent (ethanol: 20 mL, acetone: 10 mL) and three times with pure water. The resulting mesoporous polydopamine (MPDA) was obtained and freeze-dried [15, 19].

To prepare Fuziline-loaded MPDA, 5 mg of freeze-dried MPDA and 5 mg of Fuziline were resuspended in 2 mL of disodium hydrogen phosphate–citric acid buffer (pH 8.0). The mixture was subjected to ultrasonic oscillation (55 W, 4 min) using a sonicator (Sonics, USA) in an ice bath, followed by magnetic stirring at 200 rpm for 2 h. Subsequently, 200 µg of CCL2 antibody was added, and the mixture was stirred magnetically at 4 °C for 10 min. Finally, the solution was centrifuged at 4 °C and 12,000 rpm for 20 min. The resulting sediment (CFMPDA) constituted the hierarchically targeted nanoplatform. All steps were performed in the dark.

### Characterization of engineered nanoplatform

The size and zeta potential of MPDA, FMPDA, and CFMPDA were measured by dynamic light scattering (DLS, Malvern Instruments Ltd., UK). The structure and surface morphology of the nanoparticles were observed using a transmission electron microscope (TEM, Hitachi 7500, Hitachi Ltd., Tokyo, Japan). To further examine the structure of CFMPDA, high-angle annular dark field (HAADF) imaging and elemental mapping were performed using another TEM instrument (JEM 2100, JEOL Ltd., Tokyo, Japan). Ultra-performance liquid chromatography (UPLC, Waters, USA) coupled with tandem mass spectrometry (MS/MS, AB SCIEX 4000, SCIEX, Holland) was used to determine the encapsulation efficiency of Fuziline according to the following equation: Encapsulation efficiency (% w/w) = (mass of entrapped Fuziline/total initial mass of Fuziline) × 100%.

### DPPH assay

The antioxidant capacity of nanoplatforms was assessed by the DPPH radical scavenging assay. Briefly, 100 μL sample solution (0.2 mg/mL) was added to 100 µL DPPH methanol solution (DPPH: 0.1 mM) as the sample group. Meanwhile, a 100 μL the sample solution was added to 100 µl methanol as the blank group and 100 µL of H_2_O was added to 100 µl of DPPH methanol solution as the control group. The absorbance of the mixtures at 517 nm were measured after incubation for 5 min, 10 min, 20 min and 30 min sequentially. All tests were performed in 96-well microplate and protected from light at 25 ℃. Radical scavenging activity (RSA) was calculated as follows: RSA (%) = [1 − (A_sample_ − A_blank_)/A_control_] × 100, where A_control_ is the absorbance of DPPH radical + methanol, A_sample_ is the absorbance of DPPH radical + sample, and A_blank_ is the absorbance of methanol + sample [20].

### Dry eye disease model

All animal experiments were performed in accordance with relevant guidelines and regulations and were approved by the Animal Experimental Committee of Renji Hospital, Shanghai Jiao Tong University School of Medicine. The mice were housed under standard conditions with a constant temperature (22 ± 1 °C) and a 12 h light/dark cycle, with ad libitum access to food and water. Dry eye disease (DED) was induced in mice by topical application of benzalkonium chloride (BAC). Briefly, each mouse received 5 μL of 0.2% (w/v) BAC eye drops in the left eye twice daily for 14 consecutive days [5].

### Dead/living straining

Human corneal epithelial cells (HCECs) were seeded into laser confocal culture dishes at a density of 3 × 10^5^ cells/well and cultured for 24 h. The cells were then divided into five groups: mock, XOD, Fuziline, MPDA, and FMPDA. Except for the mock group, all cells were treated with XOD (70 U/L) for 24 h. Subsequently, basal medium was added to the XOD group, while the Fuziline, MPDA, and FMPDA groups received Fuziline (40 μg/mL), MPDA (40 μg/mL), and FMPDA (40 μg/mL), respectively. After 24 h of incubation, the HCECs were washed and co-stained with calcein-AM (0.67 μM) and propidium iodide (50 μg/mL) for 30 min. Live (green) and dead (red) cells were then observed and photographed using a confocal laser scanning microscope (CLSM).

### In vitro ROS detection

To evaluate the ROS scavenging ability of MPDA, HCECs (3 × 10^5^ cells/well) were cultured in six-well plates for 12 h. The cells were then divided into three groups: mock, control, and MPDA. The control and MPDA groups were sequentially treated with LPS (1 μg/mL) for 12 h and ATP (5 mM) for 1 h, followed by incubation with basal medium or MPDA (40 μg/mL) for 24 h, respectively. Untreated cells served as the mock group. After 24 h of incubation, 100 μL of DCFH-DA (1.0 mM, diluted in methanol) was added. Following incubation at 37 °C for 20 min, the cells were collected, and the percentage of ROS-positive cells was detected using a flow cytometer (Beckman).

To compare the ROS scavenging capacity of Fuziline, MPDA, and FMPDA, HCECs (3 × 10^5^ cells/well) were seeded into laser confocal culture dishes and cultured for 24 h. The cells were then divided into five groups: mock, control, Fuziline, MPDA, and FMPDA. Except for the mock group, all cells were treated with XOD (70 U/L) for 24 h. Subsequently, basal medium was added to the control group, while the Fuziline, MPDA, and FMPDA groups received Fuziline (40 μg/mL), MPDA (40 μg/mL), and FMPDA (40 μg/mL), respectively. After 24 h of incubation, 100 μL of DCFH-DA (1.0 mM, diluted in methanol) was added. After incubation at 37 °C for 20 min, the cells were observed using a confocal laser scanning microscope (CLSM, Olympus).

### Apoptotic assay

The apoptotic assay was performed using an annexin V–FITC/propidium iodide (PI) apoptosis detection kit. For verifying the anti-oxidative effects of MPDA, HCECs (3 × 10^5^) were cultured in a six-well plate and incubated at 37 °C for 12 h. Then the cells were divided into mock groups, XOD group and MPDA group, and for XOD group and MPDA group, cells were incubated with LPS (1 μg/mL) for 12 h and ATP (5 mM) for 1 h sequentially, and then were treated with basal medium and MPDA (40 μg/mL) for 24 h respectively. Untreated cells served as the mock group. At last, all cells were collected and stained, and then analyzed using flow cytometry.

To explore the anti-oxidant effects, HCECs (3 × 10^5^) were cultured in a six-well plate and incubated for 12 h. Cells were divided into five groups: mock, control, MPDA, Fuziline, and FMPDA. Cells were treated with basal medium, basal medium, Fuziline (40 μg/mL), MPDA (40 μg/mL), and FMPDA (40 μg/mL) for 4 h. The cellular supernatant of the control, MPDA, Fuziline, and FMPDA groups was then replaced with medium containing freshly diluted H₂O₂ (400 µM) to induce apoptosis. Untreated cells served as the mock group. After treatments, cells were collected, stained, and analyzed by flow cytometry.

For comparing the anti-inflammation effects of Fuziline, MPDA and FMPDA, HCECs (3 × 10^5^) were cultured in a six-well plate and incubated at 37 °C for 12 h, then all cells were treated with LPS (1 μg/mL) for 12 h and ATP (5 mM) for 1 h sequentially, and then cells were divided into four group, including control group, MPDA group, Fuziline group and FMPDA group, and were treated basal medium, Fuziline (40 μg/mL), MPDA (40 μg/mL), and MPDA (40 μg/mL) for 24 h respectively. Untreated cells served as the mock group. At last, cells were harvested and stained, and then analyzed with flow cytometry as above.

### Western blot

Whole-cell extracts were prepared using a Tissue or Cell Total Protein Extraction Kit (C510003, Sangon Biotech, China), and protein concentrations were determined by BCA assay. Subsequently, 20 μg of protein lysates were separated by SDS-PAGE. Target proteins were detected using the following antibodies: anti-Bax rabbit monoclonal antibody (1:1000, ab32503, Abcam, UK) and anti-Bcl-2 rabbit monoclonal antibody (1:10,000, ab182858, Abcam, UK). HRP-conjugated mouse monoclonal anti-GAPDH antibody (1:1000, ab9482, Abcam, UK) was used as a loading control.

### Intracellular localization of MPDA and FMPDA in vitro

The intracellular localization of MPDA and FMPDA in HCECs was assessed using confocal laser scanning microscopy (CLSM). Briefly, HCECs (5 × 10^3^ cells/well) were seeded into laser confocal culture dishes. After 12 h of incubation, the culture medium was replaced with fresh medium containing LPS (1 μg/mL). Following 8 h of induction, the medium was discarded, and the cells were washed twice with PBS. Subsequently, fresh medium containing CY5-SE-labeled MPDA or CY5-SE-labeled FMPDA (100 μg/mL) was added. The nuclei of all HCECs were stained with DAPI (Ex/Em = 364/454 nm), and mitochondria were stained with MitoTracker Green (Ex/Em = 490/516 nm). The intracellular localization of MPDA and FMPDA in HCECs was then observed and recorded using CLSM.

### Mitochondrial ROS detection

The mitochondrial ROS in HCECs was detected by CLSM and flow cytometer. HCECs were seeded into laser confocal culture dishes at a density of 3 × 10^5^ cells/well and cultured for 24 h. Then cells were divided into five group, including mock group, control group, Fuziline group, MPDA group and FMPDA group, and for control group, Fuziline group, MPDA group and FMPDA group, and cells were treated with LPS (1 μg/mL) for 12 h and ATP (5 mM) for 1 h sequentially, and then basal medium, Fuziline (40 μg/mL), MPDA (40 μg/mL), and FMPDA (40 μg/mL) were added respectively. After 24 h incubation, 100 μL of MitoSOX Red working solution were added and incubated for 20 min at 37 °C, then the Mitochondrial ROS was observed using CLSM and flow cytometer. Untreated cells served as the mock group.

### Mitochondrial morphology observation in vitro

Mitochondrial morphology was observed using transmission electron microscopy (TEM). Human corneal epithelial cells (HCECs) were seeded into 6-well plates at a density of 3 × 10^5^ cells/well and cultured for 24 h. The cells were divided into five groups: mock, control, Fuziline, MPDA, and FMPDA. Except for the mock group, all cells were sequentially treated with LPS (1 μg/mL) for 12 h and ATP (5 mM) for 1 h, followed by the addition of basal medium (control group), Fuziline (40 μg/mL), MPDA (40 μg/mL), or FMPDA (40 μg/mL), respectively. After 24 h of incubation, HCECs were collected by trypsinization and washed with PBS. The cell pellets were fixed with 2.5% glutaraldehyde overnight at 4 °C, then post-fixed with 1% osmium tetroxide for 2 h at 4 °C. The samples were subsequently dehydrated and embedded in Epon 812. Ultrathin sections were stained with uranyl acetate and lead citrate. Images were acquired using a transmission electron microscope (HT7700, Hitachi, Japan).

### Cytokine multiplex assay and bioinformatic analysis in vivo

The Invitrogen™ ProcartaPlex™ Mouse Immune Monitoring 48-Plex (EPX480-20834–901, ThermoFisher Scientific, Waltham, MA, USA) kits were employed to detect the level of inflammation factor in normal and DED corneas. Tissues were cryogenically ground, before being sonicated (4 sets of 2 s pulses, with 1 s pause between at 35% amplitude) in ProcartaPlex lysis buffer (EPX 99999–001, ThermoFisher Scientific, Waltham, MA, USA), then normalized to 1.2 mg mL^−1^ using Bradford assay. For all kits, the manufacturer’s instructions were followed during assay, using a Biotek 405 ts magnetic plate washer. Briefly, beads were added to a black clear-bottom 96-well plate and incubated with samples and a dilution series of standards overnight. After being washed, the beads were then incubated with primary antibodies, washed, incubated with secondary antibodies, washed, then resuspended in a final reading solution. Plates were read using a Luminex 200 System [21]. For data profiles, cytokine interaction networks were constructed using the STRING database (confidence score ≥ 1000). Degree centrality identified top 10 hub cytokines, which were further analyzed via Ingenuity Pathway Analysis (IPA) – Predicted functional interactions and regulatory pathways and KEGG Pathway Enrichment – Mapped hub cytokines to biologically relevant pathways.

### Histopathological analysis

Following treatment with CCL2 antibody, Fuziline, and MPDA on DED mice, the animals were euthanized, and their corneal and conjunctival tissues were harvested and divided into three segments. The samples underwent H&E staining, Masson's trichrome staining, and TUNEL staining. For immunofluorescence analysis, the fixed and dehydrated corneal and conjunctival sections were blocked and then incubated with primary antibodies at 4 °C overnight. Subsequently, the slides were treated with corresponding secondary antibodies at 37 °C for 1 h in the dark. Finally, the sections were mounted with DAPI Fluoromount-G® (Southern Biotech, AL, USA) and visualized under a fluorescence microscope.

### RNA sequencing transcription and analysis

High-throughput RNA sequencing was performed to investigate changes in corneal mRNA expression following CCL2 antibody treatment in a dry eye disease (DED) model. Total RNA was extracted from corneas using TRIzol reagent (15,596,026; Ambion, Austin, TX, USA), and RNA integrity was assessed on an Agilent 2100 Bioanalyzer (Thermo Fisher Scientific, Waltham, MA, USA). Samples with an RNA integrity number (RIN) ≥ 7 were used for subsequent analysis. After cDNA library construction, transcriptome sequencing was conducted on an MGISEQ-2000 platform, and data analysis was performed using the Dr. Tom platform (http://report.bgi.com; BGI, Shenzhen, China). This approach enabled the identification of mRNA expression changes in corneas after Fuziline treatment in the DED model.

### In vivo fluorescence imaging and quantification

The dispersion and retention of FMPDA and CFMPDA in the ocular anterior segment of normal and dry eyes were evaluated using an IVIS Lumina III in vivo imaging system (PerkinElmer, Waltham, MA, USA). Briefly, dry eye disease (DED) was induced in mice by administering BAC for 0 (control), 7, and 14 days. A separate treatment group received CFMPDA therapy starting after 14 days of BAC treatment and continued for 5 days. Once the DED model was established, each group was further divided into two subgroups that received 5 μL of CY5-SE-labeled FMPDA (1 mg/mL) or CY5-SE-labeled CFMPDA (1 mg/mL) eye drops, respectively. The mice were then anesthetized, and fluorescence signals on the ocular surface were monitored using the IVIS system at 5, 10, 20, 30, 45, and 60 min post-administration. At 60 min, the mice were euthanized by an overdose of pentobarbital (150 mg/kg), and the eyeballs were collected for ex vivo fluorescence imaging.

### Intracellular localization of MPDA, FMPDA and CFMPDA in vivo

The intracellular internalization of MPDA, FMPDA, and CFMPDA in corneal epithelial cells was evaluated using confocal laser scanning microscopy (CLSM). Briefly, following successful induction of the dry eye disease (DED) model by treatment with BAC for 7 days, all animals were divided into three groups: the MPDA group, the FMPDA group, and the CFMPDA group, which received 5 μL of CY5-SE-labeled MPDA, CY5-SE-labeled FMPDA, and CY5-SE-labeled CFMPDA eye drops, respectively. Immediately after administration, the mice were kept in a dark environment. Two hours later, the mice were sacrificed, and the corneas were immediately dissected and prepared as 7 μm frozen sections. The sections were then fixed, blocked, and stained with MitoTracker Green and DAPI. All sections were visualized using CLSM.

### Mitochondrial morphology observation in vivo

Following treatment with Fuziline, MPDA, FMPDA, and CFMPDA in DED mice for five days, all mice were euthanized, and their corneal tissues were immediately dissected and washed with PBS. The tissues were fixed with 2.5% glutaraldehyde overnight at 4 °C. Untreated corneal tissues served as the mock control. The specimens were then post-fixed with 1% osmium tetroxide for 2 h at 4 °C, dehydrated through a graded ethanol series, and embedded in Epon 812. Ultrathin sections were stained with uranyl acetate and lead citrate. Images were acquired using a transmission electron microscope (HT7700, Hitachi, Japan).

### Comparison of the therapeutic effects of fluorometholone and CFMPDA on DED

After successful establishment of the DED mouse model, the animals were divided into two groups: the Fluorometholone group and the CFMPDA group. The mice received respective treatments (Fluorometholone: 10 μL; CFMPDA: 300 μg/mL, 10 μL) three times daily. Fluorescein sodium staining was performed daily to assess corneal damage. After five days, all mice were euthanized, and conjunctival and corneal tissues were collected for molecular biology and histopathological analysis.

### Statistical analysis

All quantitative data were presented as the means ± SD. Statistical analysis was performed with GraphPad Prism version 8.3.0 (GraphPad Software, CA, USA) and IBM SPSS Statistics 29 (IBM Corporation, Armonk, NY) through multiple *T*-test, row mean with SD, Simple linear regression, ordinary one-way ANOVA, two-way ANOVA and three-way ANOVA. Statistical significance was set to *P* < 0.05.

## Results and discussion

### Rational design of a hierarchical nanotherapeutic guided by pathological mapping

**Fig. 2:**
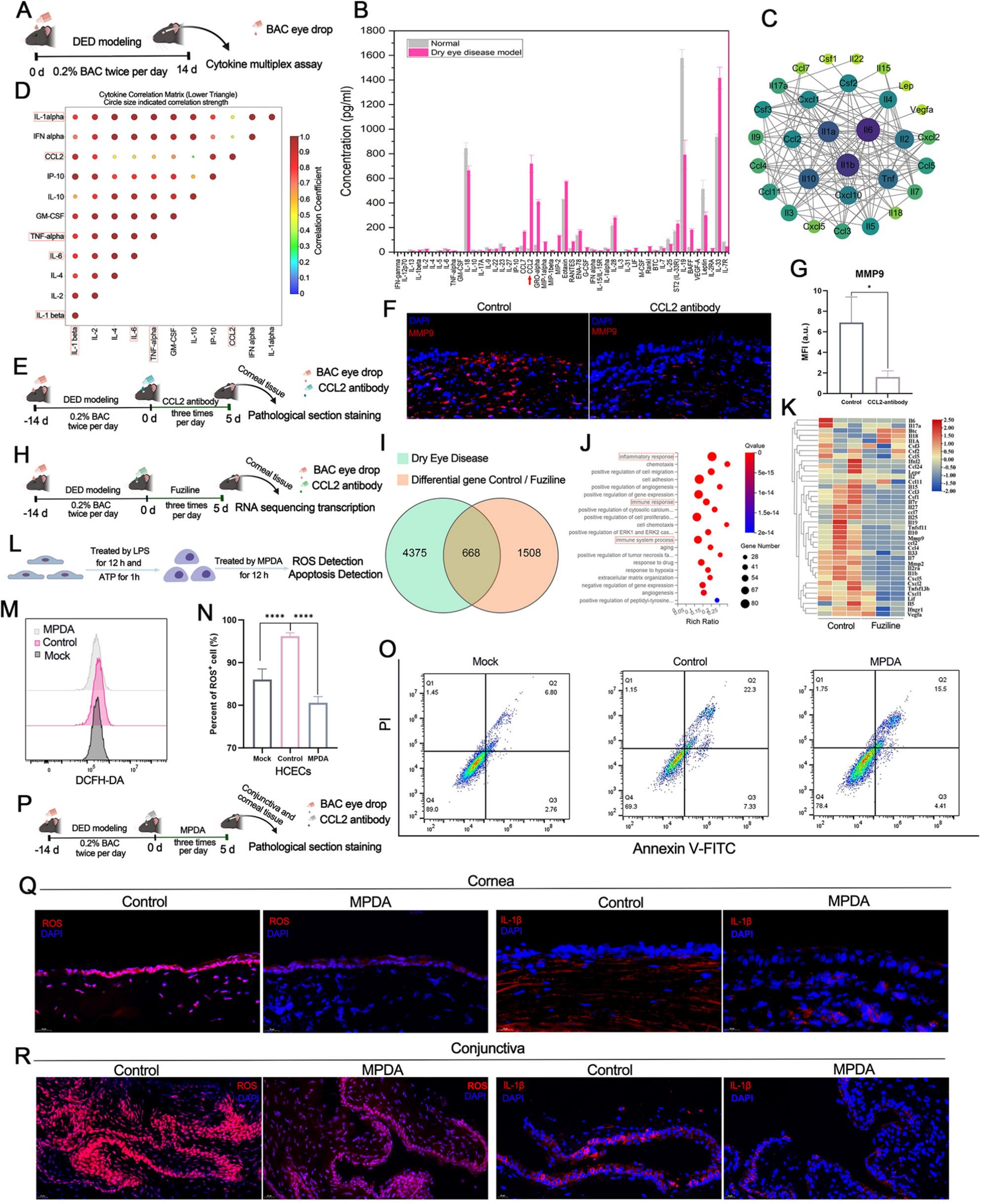
The effects of CCL2, Fuziline, and MPDA on dry eye disease (DED). (**A**) Schematic illustration of the experimental procedure for the cytokine multiplex assay. (**B**) Concentrations of 48 cytokines in normal and DED corneas. (**C**) STRING protein–protein interaction network of cytokines with a confidence score ≥1000. (**D**) Heatmap showing interactions among the top 11 hub cytokines. (**E**) Schematic of in vivo DED modeling and subsequent treatment with CCL2 antibody. (**F**) Representative immunofluorescence images of MMP9 in corneal sections. (**G**) Quantification of the mean fluorescence intensity (MFI) of MMP9 in corneal sections. (**H**) Schematic of in vivo DED modeling and subsequent treatment with Fuziline. (**I**) Venn diagram showing overlapping targets between DED-associated genes from the GeneCards database and differentially expressed corneal genes (control vs. Fuziline groups). (**J**) KEGG enrichment analysis of the overlapping genes. (**K**) Heatmap showing the RNA expression levels of key corneal cytokines in the control and Fuziline groups. (**L**) Schematic illustration of the experimental procedure for evaluating MPDA-mediated ROS scavenging and apoptosis suppression in HCECs. (**M**) Flow cytometric curves showing ROS levels in HCECs. (**N**) Quantitative analysis of flow cytometry data. (**O**) Flow cytometry analysis of apoptosis in HCECs after various treatments. (**P**) Schematic of in vivo DED modeling and subsequent treatment with MPDA. (**Q**, **R**) Representative immunofluorescence images of ROS and IL-1β in corneal and conjunctival sections. Data are presented as mean ± SD (n = 3). Significance was set at *P < 0.05, **P < 0.01, ***P < 0.001, and ****P < 0.0001. Statistical analyses were performed using two-tailed Student's t-test (**G**) and one-way ANOVA (**N**)

To identify a master regulator that could strategically disrupt the inflammatory cascade in DED and serve as a reliable targeting anchor for pathological tissues, we employed an unbiased proteomic screening. Using a cytokine multiplex assay on corneal tissues from a well-established DED mouse model (Fig. 2A), we found that, among 48 cytokines analyzed, CCL2 exhibited a most pronounced upregulation (Fig. 2B). This striking elevation immediately positioned CCL2 as a potential key node in the disease pathogenesis. To decode the functional relevance of CCL2 overexpression, we performed integrative bioinformatics analysis. Protein–protein interaction (PPI) network mapping of the dysregulated cytokines revealed that CCL2 occupies a central position, directly interacting with core pro-inflammatory cytokines (IL-1β, IL-6, TNF-α) (Fig. 2C). More importantly, correlation analysis demonstrated strong positive correlations (coefficient > 0.5) between CCL2 and these effector cytokines (Fig. 2D), suggesting a coordinated upregulation and implicating CCL2 not merely as a bystander but as a likely driver of the broader inflammatory response. KEGG pathway analysis further fortified this notion, linking CCL2 to the IL-17 signaling pathway—a known amplifier of IL-1β, IL-6, TNF-α, and MMP9 expression (Fig. S1A) [22]. To verify these findings at the cellular level, human corneal epithelial cells (HCECs) were induced with LPS + ATP, and changes in cytokine levels in the cell supernatants were analyzed. The results were consistent with the in vitro findings (Fig. S1 B-E).

Having established CCL2’s central role in silico and in vivo, we sought to directly test its therapeutic potential. Administration of a CCL2-neutralizing antibody in DED mice (Fig. 2E) led to a significant reduction in corneal infiltration of monocytes (Fig. S2G), confirming the effective blockade of CCL2’s chemoattractant function. Crucially, this intervention yielded profound therapeutic benefits: corneal epithelial damage (assessed by fluorescein staining) was markedly alleviated (Fig. S2A) [23], levels of the destructive protease MMP9 were reduced (Fig. 2F and G), and infiltration of pathogenic T cells (CD3⁺, CD4⁺, IL-23R⁺) into the cornea was significantly suppressed (Fig. S2) [24]. These findings were further corroborated by transcriptomic profiling, which revealed coordinated downregulation of immune-related pathways and inflammatory markers (Fig. S2 B, C, E, F, H, I and J). Collectively, these data establish CCL2 as a lynchpin within the DED inflammatory network. Its inhibition successfully attenuates multiple downstream pathological events—from cytokine production and MMP activation to immune cell recruitment—thereby validating it as a potent and strategic anchor for targeted drug delivery to the inflamed ocular surface.

To complement the precise, upstream blockade provided by a CCL2 antibody, we next sought a broad-spectrum anti-inflammatory agent capable of suppressing the downstream cascade. We focused on Fuziline, a natural diterpenoid alkaloid with reported anti-inflammatory properties. To first evaluate its cytoprotective efficacy, we treated LPS + ATP-stimulated human corneal epithelial cells (HCECs) with Fuziline. The CCK-8 assay confirmed that Fuziline significantly restored cell viability, with an optimal concentration of 30 μg/mL (Fig. S3A). To gain a systems-level understanding of its mechanism, we performed unbiased transcriptomic RNA sequencing on corneas from DED mice treated with Fuziline (Fig. 2H). This analysis revealed that Fuziline treatment modulated a substantial set of 2176 genes. Intersecting these with known DED-associated genes from the GeneCards database identified 668 overlapping targets (Fig. 2I), strongly indicating a direct action on the disease’s molecular network. Kyoto Encyclopedia of Genes and Genomes (KEGG) enrichment analysis of these overlapping genes highlighted pathways central to “inflammatory response,” “immune response,” and “immune system process” (Fig. 2J) [25].

Crucially, at the individual gene level, Fuziline treatment led to the coordinated downregulation of key mediators across the entire inflammatory axis. This included pro-inflammatory cytokines (e.g., IL‐1β, IL‐6), chemotaxis-related enzymes (e.g., MMP9), and markers associated with T cell activation and function (e.g., CD3g, CD4, Il17r) (Figs. 2K, S3C). Notably, Fuziline also downregulated the transcription of CCL2 itself in inflamed HCECs (Fig. S3B), suggesting it can suppress the expression of our primary target. The therapeutic relevance was confirmed in vivo, where Fuziline treatment significantly alleviated corneal epithelial damage in DED mice (Fig S3D and E). In summary, Fuziline is not a single-target agent but a multifaceted anti-inflammatory hub. Its broad-spectrum activity profile makes it an ideal therapeutic payload to synergize with the precise, upstream targeting provided by a CCL2 antibody.

To simultaneously address the oxidative stress arm of DED pathology, we engineered a nanomaterial core with intrinsic therapeutic activity, aiming to achieve subcellular functional delivery. We selected mesoporous polydopamine (MPDA), leveraging its well-documented free radical scavenging capacity and versatile drug-loading capability [26]. We first rigorously confirmed its antioxidant prowess in vitro. In HCECs subjected to LPS and ATP in sequence (Fig. 2L), MPDA treatment dramatically reduced the population of ROS⁺ cells from 96.18% to 80.57% and, more importantly, lowered the apoptotic cell percentage from 26.42% to 8.62% (Figs. 2M**-**O, S4C).

The therapeutic potential of MPDA's standalone antioxidant activity was then validated in the complex DED milieu. In DED mice, treatment with MPDA alone led to significant mitigation of ocular surface damage, evidenced by reduced corneal fluorescein staining (Figs. 2P, S4A and B). Critically, histological and immunofluorescence analyses confirmed that MPDA effectively scavenged excess ROS and reduced IL-1β levels not only in the cornea but also in the conjunctiva (Figs. 2Q and R, S4C and D). Furthermore, TUNEL staining revealed a significant decrease in apoptosis in both corneal and conjunctival epithelia upon MPDA treatment (Fig. S4F-I). Beyond its role as an active therapeutic (“nanoscavenger”), the mesoporous structure of MPDA was strategically utilized as a drug delivery platform. We successfully loaded Fuziline into its pores with high efficiency (49.04 ± 2.52%, Fig. S2E), creating a combined therapeutic entity (FMPDA). This design transforms MPDA from a mere carrier into a dual-function core: it directly neutralizes pathogenic ROS while controllably releasing an anti-inflammatory payload.

Based on these foundational insights, we embarked on a rational nano-engineering endeavor to integrate these components into a unified, multifunctional platform. The compelling evidence of CCL2’s pivotal role and its overexpression in the diseased milieu (Fig. 2B) provided a clear pathology-guided targeting strategy. We postulated that conjugating CCL2 antibodies onto nanoplatforms would enable active recognition and binding to the inflamed ocular surface, thereby fundamentally altering their pharmacokinetics. This design embodies a smart, feedback-loop characteristic: the nanoplatform’s retention is actively enhanced at the disease site where CCL2 is abundant, while its clearance is facilitated as inflammation resolves and CCL2 levels decline. This pathology-responsive behavior is engineered to maximize therapeutic exposure at the target while minimizing off-target accumulation, addressing a key challenge in ocular drug delivery. Furthermore, we engineered the platform not as a simple mixture but as a synergistically integrated system. The mesoporous polydopamine (MPDA) core serves a dual purpose: as a nanoscale reservoir for the sustained release of the broad-spectrum anti-inflammatory agent Fuziline, and as an intrinsically active component for scavenging ROS. This spatiotemporally coordinated combination—CCL2-mediated anchoring, Fuziline-driven anti-inflammation, and MPDA-enabled antioxidant action—is designed to launch a concerted attack on the intertwined inflammatory-oxidative cycle of DED (Fig. 1).

Collectively, this rational design translates our mechanistic discoveries into a sophisticated nanotherapeutic agent, aiming to achieve targeted, synergistic, and condition-adaptive therapy for DED.

### Synthesis and tailored characterization of a multifunctional nanoplatform

**Fig. 3:**
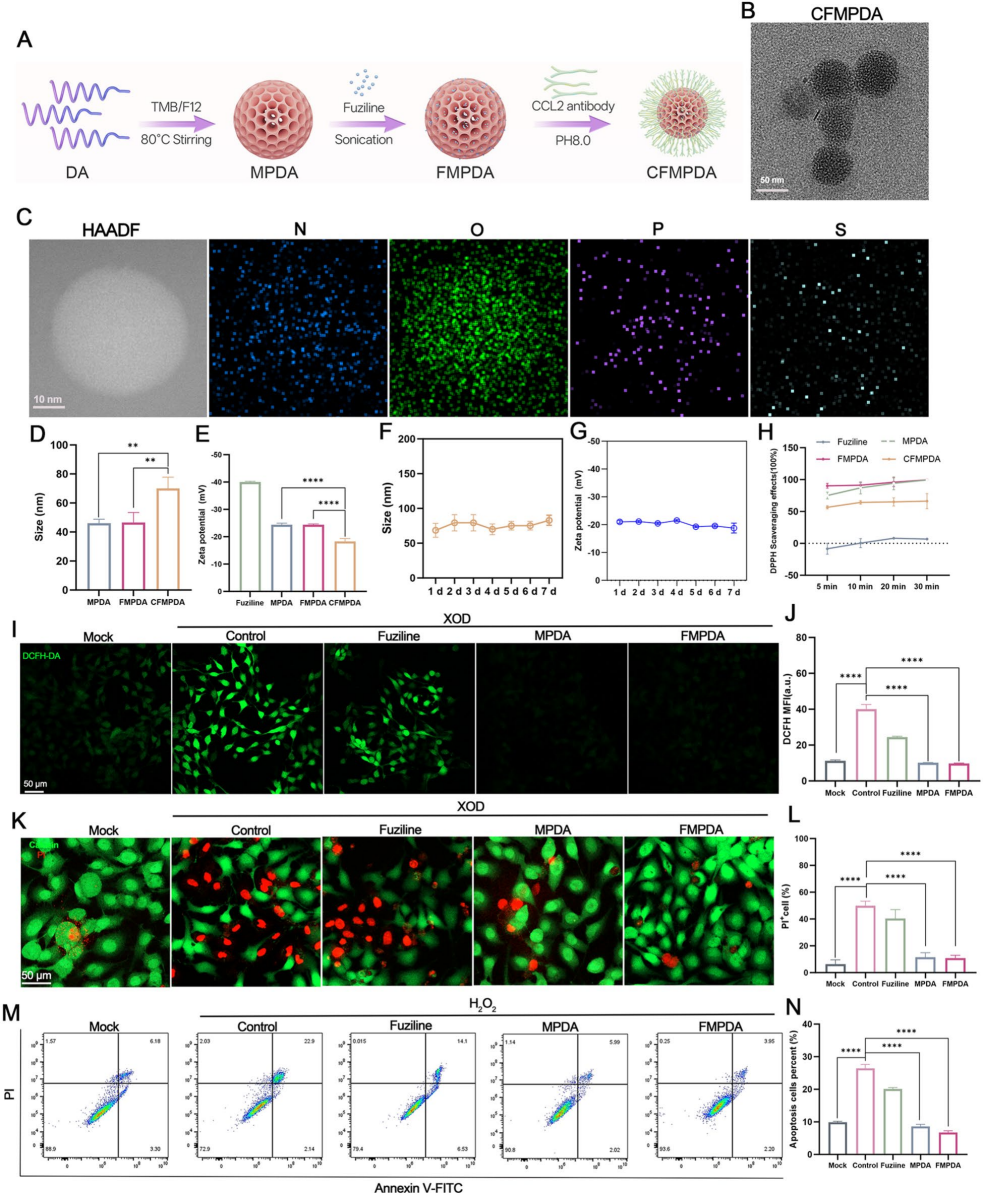
In vitro characterization of the antioxidant properties of nanoplatforms. (**A**) Schematic illustration of the synthesis process of MPDA, FMPDA, and CFMPDA. (**B, C**) TEM image and elemental mapping images of CFMPDA. (**D**) Size distribution of MPDA, FMPDA, and CFMPDA. (**E**) Zeta potential of fuziline, MPDA, FMPDA, and CFMPDA. (**F**) Size distribution of CFMPDA over time. (**G**) Zeta potential of CFMPDA over time. (**H**) DPPH radical scavenging activity of fuziline, MPDA, FMPDA, and CFMPDA. (**I, J**) Representative CLSM images of HCECs stained with DCFH-DA after different treatments and the corresponding quantification of mean fluorescence intensity (MFI) showing intracellular ROS levels. (**K, L**) Representative CLSM images of HCECs stained with PI and calcein after different treatments and the corresponding MFI quantification. (**M**) Flow cytometry analysis of H₂O₂-induced apoptosis in HCECs after incubation with fuziline, MPDA, and FMPDA. (**N**) Quantitative analysis of flow cytometry data. Data are presented as mean ± SD (n = 3). Significance was set at *P < 0.05, **P < 0.01, ***P < 0.001, and ****P < *0.0001*. Statistical analyses were performed using one-way ANOVA (**D**, **E**, **F**, **G**, **J**, **L**, **N**)

Guided by the therapeutic rationale established above, we proceeded with the precise engineering of our sequential-targeting nanoplatform. The synthesis followed a modular, stepwise strategy to integrate distinct functionalities (Fig. 3A). First, monodisperse mesoporous polydopamine nanoplatforms (MPDA) were synthesized via a templating method, providing a high-surface-area core with inherent antioxidant capacity. This core was then functionalized by loading the anti-inflammatory drug Fuziline through a diffusion process, yielding FMPDA. The final, key step involved the covalent conjugation of CCL2 antibodies to the surface of FMPDA via the reactive quinone groups on polydopamine, resulting in the complete CFMPDA nanoconstruct.

Comprehensive physicochemical characterization confirmed the successful stepwise assembly and revealed critical structure–property relationships. Transmission electron microscopy (TEM) imaging showed that all nanoparticles maintained a spherical morphology throughout the functionalization steps. Notably, after antibody conjugation, CFMPDA exhibited a discernible surface coating not present on MPDA or FMPDA (Figs. 3B, S5B). Elemental mapping provided direct evidence for this surface modification, confirming the uniform presence of sulfur (S), an element characteristic of antibodies, exclusively on CFMPDA (Fig. 3C). Dynamic light scattering (DLS) analysis revealed a controlled increase in hydrodynamic diameter from 46.08 ± 2.76 nm (MPDA) to 46.57 ± 6.88 nm (FMPDA) and finally to 70.07 ± 7.74 nm for CFMPDA (Fig. 3D). This significant size increase for CFMPDA is attributed to the successful attachment of the antibody layer. Consistent with this, the surface zeta potential became less negative, shifting from −24.36 ± 0.61 mV (MPDA) to −18.3 ± 1.05 mV (CFMPDA), due to the partial shielding of the negatively charged PDA surface by the proteinaceous antibodies (Fig. 3E).

We further quantified the drug loading capacity and evaluated the colloidal stability and release behavior of the final construct. Quantitative analysis via UPLC-MS/MS determined a high Fuziline encapsulation efficiency of 49.04 ± 2.52% in CFMPDA (Fig. S5E), confirming the efficacy of MPDA as a nanocarrier. Critically, the engineered CFMPDA demonstrated excellent colloidal stability in physiologically relevant phosphate-buffered saline (PBS) over 7 days, with negligible changes in both size and zeta potential (Fig. 3F and G). This stability is essential for reliable performance in subsequent in vitro and in vivo applications.

To assess drug delivery control, we further investigated the in vitro release kinetics of Fuziline from the nanoplatforms. As shown in Fig. S5F, FMPDA exhibited a rapid release profile, reaching 93.5 ± 3.5% cumulative release at 72 h. In striking contrast, CFMPDA demonstrated a significantly slower and more sustained release, with only 22.7 ± 3.4% cumulative release over the same period. This marked difference is attributed to the steric hindrance conferred by the surface-conjugated CCL2 antibody, which forms a physical barrier that impedes the diffusion of Fuziline from the mesoporous core into the release medium. These data confirm that antibody conjugation not only confers active targeting capability but also modulates drug release kinetics, endowing CFMPDA with enhanced sustained-release properties that are highly desirable for long-term therapeutic intervention in DED.

In summary, we have successfully fabricated a hierarchically structured nanoplatform through a controlled, multi-step synthesis. The systematic characterization verifies the integration of all designed components—the antioxidant MPDA core, the encapsulated Fuziline, and the surface-anchored targeting antibodies—resulting in a stable, well-defined nanoplatform with the tailored physicochemical properties necessary for its proposed sequential targeting mission.

### The nanoplatform functions as an active nanotherapeutic via ROS scavenging and mitochondrial protection

Having established the successful synthesis and structural integrity of our nanoplatforms, we next sought to rigorously quantify their intrinsic bioactivity as reactive oxygen species (ROS) scavengers—a cornerstone of their designed therapeutic function. We first assessed their inherent radical quenching capacity using a chemical antioxidant assay. The 2,2-diphenyl-1-picrylhydrazyl (DPPH) assay revealed that while free Fuziline showed minimal activity (6.72 ± 0.54% inhibition), both MPDA and FMPDA demonstrated near-complete radical scavenging (> 99% inhibition) at 100 µg/mL (Fig. 3H). Significantly, the final construct, CFMPDA, retained substantial activity (66.33 ± 11.76% inhibition), confirming that the conjugation of targeting antibodies does not compromise the core antioxidant function derived from the MPDA scaffold. To evaluate this bioactivity in a more complex, biologically relevant context, we modeled oxidative stress in human corneal epithelial cells (HCECs). We employed xanthine oxidase (XOD) to induce sustained intracellular ROS generation [27]. Confocal microscopy visually confirmed this robust ROS scavenging capability, as evidenced by markedly diminished DCFH-DA fluorescence in cells treated with the nanoplatforms compared to the stressed control (Fig. 3I and J).

We further investigated the downstream functional consequences of ROS removal on critical cellular health parameters. Live/Dead staining demonstrated that MPDA and FMPDA treatment cut the population of dead (PI⁺) cells by more than 75%, from 49.9% in the control to ~11% (Fig. 3K and L). Crucially, this cytoprotection was linked to the preservation of mitochondrial health, a primary target of oxidative damage. JC-1 staining showed that MPDA and FMPDA effectively maintained mitochondrial membrane potential (ΔΨm), with JC-1 red/green ratios above 1.0, in stark contrast to the collapsed potential (ratio ~0.4) in stressed control cells (Fig. S6A). This mitochondrial restoration was further corroborated by reduced staining with dihydroethidium (DHE), a superoxide indicator (Fig. S6B). The ultimate cellular outcome of this comprehensive antioxidant defense was a profound suppression of apoptosis. Flow cytometric analysis using Annexin V/PI staining revealed that the apoptotic cell fraction was reduced approximately 3- to fourfold in the MPDA and FMPDA groups (8.62% and 6.72%, respectively) compared to the H₂O₂-induced control (26.42%) (Fig. 3M and N).

Collectively, these in vitro data unequivocally establish that the MPDA core is not a passive carrier but an intrinsically active nanoscavenger. Its integration into the FMPDA and CFMPDA constructs confers upon them a powerful, multi-faceted cytoprotective ability: directly neutralizing free radicals, safeguarding mitochondrial integrity, and thereby preventing ROS-induced apoptotic cell death. This robust antioxidant foundation is essential for the platform’s proposed mechanism to disrupt the oxidative arm of the DED pathological cycle at the subcellular level.

### Active targeting enables pathology-responsive pharmacokinetics

**Fig. 4:**
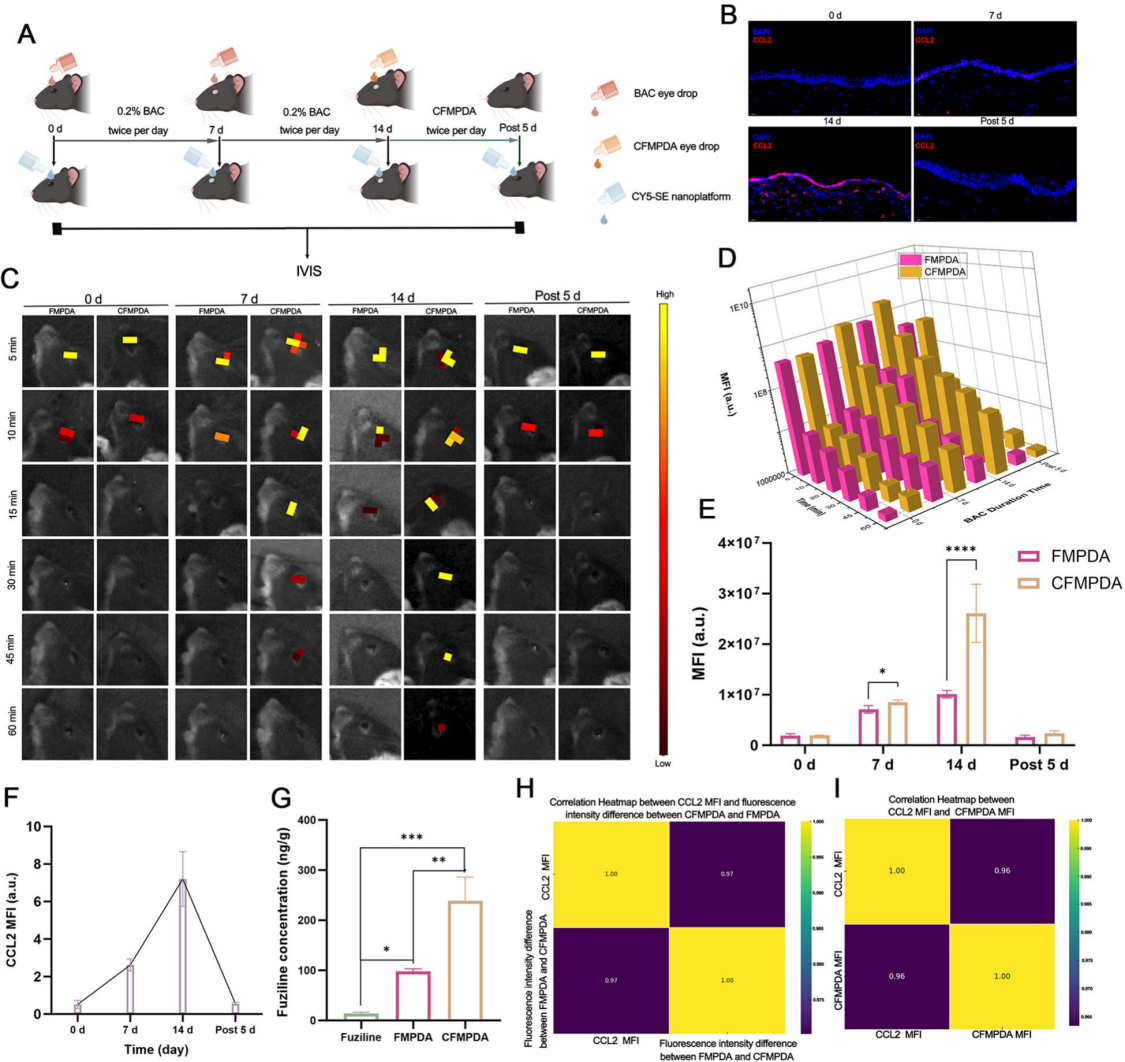
CCL2-targeting behavior of nanoplatforms and their ocular retention. (**A**) Schematic illustration of in vivo DED modeling and evaluation of the dispersion and retention of FMPDA and CFMPDA in the ocular anterior segment of normal and DED eyes after different durations of BAC treatment. (**B, F**) Representative immunofluorescence images of CCL2 in corneal sections and the corresponding MFI quantification. (**C**) In vivo distribution of CY5-SE-labeled FMPDA and CFMPDA in mice treated with BAC. Mice received a single eye drop of CY5-SE-labeled FMPDA or CFMPDA at different time points during BAC treatment. (**D**) 3D plot showing MFI quantification from IVIS imaging. (**E**) Quantification of fluorescence signals in ex vivo eyeballs. (**G**) Fuziline concentration in corneas (after 14 days of BAC treatment) following administration of fuziline, FMPDA, or CFMPDA into the conjunctival sac. The initial dose of Fuziline from each eye drop formulation was 1.5 mg/kg. (**H**) Heatmap showing the correlation between CCL2 fluorescence intensity and the difference in fluorescence signal between FMPDA and CFMPDA (60 min after CY5-SE-labeled eye drop administration) at different BAC treatment durations. (**I**) Heatmap showing the correlation between CCL2 fluorescence intensity and CFMPDA MFI (60 min after CY5-SE-labeled eye drop administration) at different BAC treatment durations. Data are presented as mean ± SD (n = 3). Significance was set at *P < 0.05, **P < 0.01, ***P < 0.001, and ****P < *0.0001*. Statistical analyses were performed using two-tailed Student's t-test (E) and one-way ANOVA (G)

A cornerstone of our design is the active, pathology-responsive targeting conferred by surface-conjugated CCL2 antibodies. To validate our central hypothesis—that anti-CCL2 conjugation enables active, pathology-dependent targeting and fundamentally alters ocular pharmacokinetics—we performed quantitative in vivo biodistribution studies. We first established a robust correlation between the fluorescence intensity of CY5-SE-labeled nanoplatforms and their concentration in vitro (R^2^ > 0.99, Fig. S7A-D), ensuring that in vivo signal could be reliably interpreted as nanoplatform quantity. We then systematically investigated the ocular surface retention of non-targeted (FMPDA) versus targeted (CFMPDA) nanoplatforms across a spectrum of disease states. DED mouse models with varying severity—induced by 0, 7, or 14 days of benzalkonium chloride (BAC) treatment—were established (Fig. 4A). Immunofluorescence confirmed that corneal CCL2 expression increased with disease progression, peaking at 14 days (MFI: 7.21 ± 0.46) and returning to near-baseline levels after a 5-day recovery period (Fig. 4B and F).

The in vivo imaging results revealed a striking, disease-severity-dependent retention profile exclusive to the targeted construct. Following topical administration, the fluorescence signal of CFMPDA decayed significantly more slowly than that of FMPDA, particularly at the 7- and 14-day disease time points (Figs. 4C and D, S7H-J). Ex vivo analysis of enucleated eyeballs 60 min post-administration quantified this difference: CFMPDA exhibited significantly higher residual fluorescence than FMPDA specifically in inflamed eyes (7- and 14-day groups), but not in healthy (0-day) or recovered (Post-5-day) eyes (Figs. 4E, S7E). This precise correlation between enhanced retention and pathological CCL2 elevation strongly implicates the antibody-antigen interaction as the governing mechanism.

A rigorous correlation analysis cemented this relationship. The difference in fluorescence retention between CFMPDA and FMPDA showed an exceptionally strong positive correlation with corneal CCL2 levels (correlation coefficient = 0.97, Fig. 4H). Similarly, the absolute fluorescence intensity of CFMPDA alone correlated strongly with CCL2 expression (coefficient = 0.96, Fig. 4I). These data quantitatively demonstrate that our nanoplatform’s ocular surface residence time is dynamically regulated by the pathological biomarker it targets, embodying a “sense-and-respond” behavior highly desired in smart drug delivery. This behavior ensures the carrier’s pharmacokinetics are dictated by the disease state, maximizing exposure at the target while minimizing off-target accumulation.

The functional consequence of this enhanced retention was a dramatic improvement in corneal drug bioavailability. Pharmacokinetic analysis 60 min post-administration in severely inflamed (14-day) corneas showed that the Fuziline concentration delivered by CFMPDA was approximately tenfold and 2.4-fold higher than that delivered by free Fuziline and non-targeted FMPDA, respectively (Figs. 4G, S7G). This confirms that the CCL2-targeting strategy is not merely a binding event but a functional mechanism to actively promote the localized enrichment of the therapeutic payload within the diseased tissue, effectively overcoming the first major barrier (rapid precorneal clearance) in ocular drug delivery.

### Programmed intracellular trafficking and mitochondrial remediation

Following successful disease-site anchoring, a sophisticated delivery system must navigate intracellular barriers to reach the relevant pathological organelles. We investigated the programmed intracellular mission of our nanoplatform—its ability to localize to and remediate mitochondria, the epicenter of oxidative damage. We first confirmed that inflamed corneal epithelial cells (HCECs) efficiently internalized both MPDA and FMPDA nanoplatforms, with uptake exceeding 95% after LPS stimulation, independent of Fuziline loading (Fig. S8A-C).

**Fig. 5:**
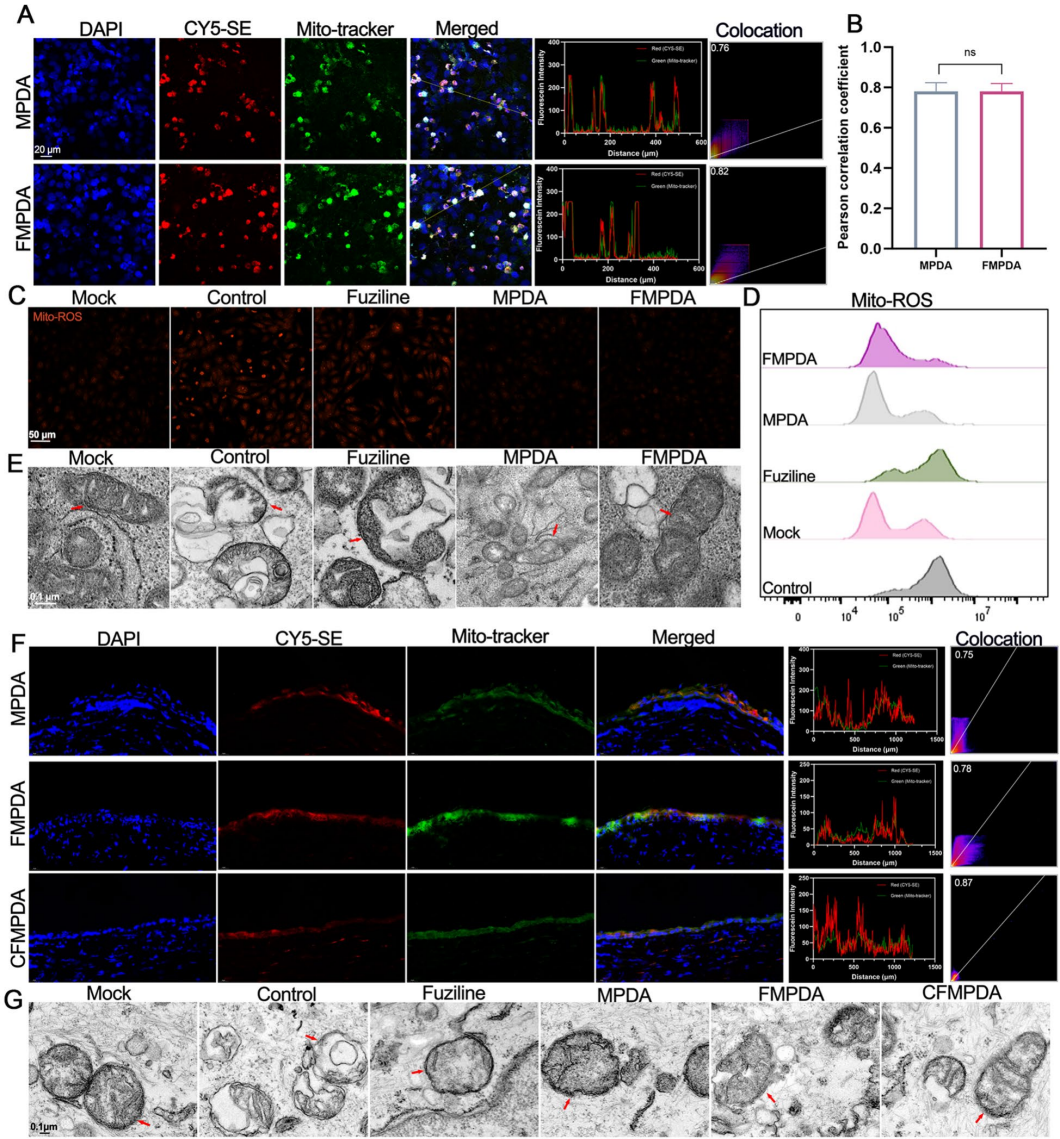
Mitochondrial colocalization of nanoplatforms. (**A**) Intracellular localization of MPDA and FMPDA in HCECs. Cells were induced with LPS and ATP, then incubated with CY5-SE-labeled MPDA or FMPDA for 4 h. (**B**) Pearson correlation coefficients for MPDA and CFMPDA colocalization. Data are shown as mean ± SD. Statistical analysis was performed using a two-tailed Student's t-test. (**C**) Representative CLSM images of HCECs stained with MitoSOX after different treatments. (**D**) Flow cytometric curves showing MitoSOX levels in HCECs. (**E**) Representative TEM images of mitochondria in HCECs after various treatments. Red arrows indicate mitochondria. (**F**) Intracellular localization of MPDA, FMPDA, and CFMPDA in corneal sections. (**G**) Representative TEM images of mitochondria in corneal epithelial cells after various treatments. Red arrows indicate mitochondria. Data are presented as mean ± SD (n = 3). Significance was set at *P < 0.05, **P < 0.01, ***P < 0.001, ****P < *0.0001*, and ns = no significance. Statistical analysis in (**B**) was performed using a two-tailed Student's t-test

Critical confocal microscopy analysis revealed the precise subcellular destination. Following cellular uptake, Cy5-labeled MPDA or FMPDA nanoplatforms exhibited significant co-localization with MitoTracker Green, evidenced by high Pearson’s correlation coefficients (~0.78) (Fig. 5A and B). This confirms that the intrinsic physicochemical properties of the MPDA core are sufficient to direct the nanoplatforms to mitochondria post-internalization, a function preserved in the FMPDA construct. This organelle-specific tropism is a critical design feature, ensuring that the therapeutic antioxidant activity is delivered precisely to the primary intracellular source of ROS.

We then assessed the functional consequence of this mitochondrial localization by measuring organelle-specific ROS. In HCECs subjected to LPS + ATP-induced inflammatory-oxidative stress, treatment with MPDA or FMPDA drastically reduced mitochondrial superoxide levels, as shown by diminished MitoSOX Red fluorescence in both CLSM and flow cytometry (Mito-ROS⁺ cells: from ~83.5% to ~28%, Figs. 5C and D, S8D). This demonstrates that the nanoplatforms actively scavenge ROS at their primary intracellular generation site.

The ultimate proof of functional rescue was observed at the ultrastructural level. Transmission electron microscopy (TEM) of HCECs and, importantly, of corneal epithelial cells from DED mice, provided direct visual evidence. Cells and tissues treated with MPDA or FMPDA preserved normal mitochondrial morphology with distinct cristae, in stark contrast to the swollen, vacuolized mitochondria observed in stressed control groups (Fig. 5E and G). This restoration of mitochondrial architecture is a hallmark of recovered bioenergetic health and cellular viability, and serves as definitive proof of functional intracellular delivery.

Strikingly, this mitochondrial targeting and functional remediation was fully retained in the complete, sequentially-targeting construct, CFMPDA. In the DED mouse model, CFMPDA administered topically was internalized by corneal epithelial cells and demonstrated identical mitochondrial co-localization (Pearson’s coefficient ~0.82) as its precursors (Figs. 5F, S8E). This confirms that the surface conjugation of the CCL2 antibody, responsible for the first targeting step, does not interfere with the MPDA core’s inherent ability to execute the second targeting step to mitochondria.

In summary, these data complete the functional validation of our hierarchical targeting strategy. The nanoplatform successfully navigates from the macroscopic disease site (CCL2⁺ ocular surface) to the microscopic pathological organelle (dysfunctional mitochondria). By directly delivering its ROS-scavenging capability to the mitochondria, it quenches oxidative stress at its source, rescues organelle integrity, and fulfills a critical mechanism for breaking the DED cycle.

### Synergistic therapeutic outcomes validate the integrated sequential targeting strategy

**Fig. 6:**
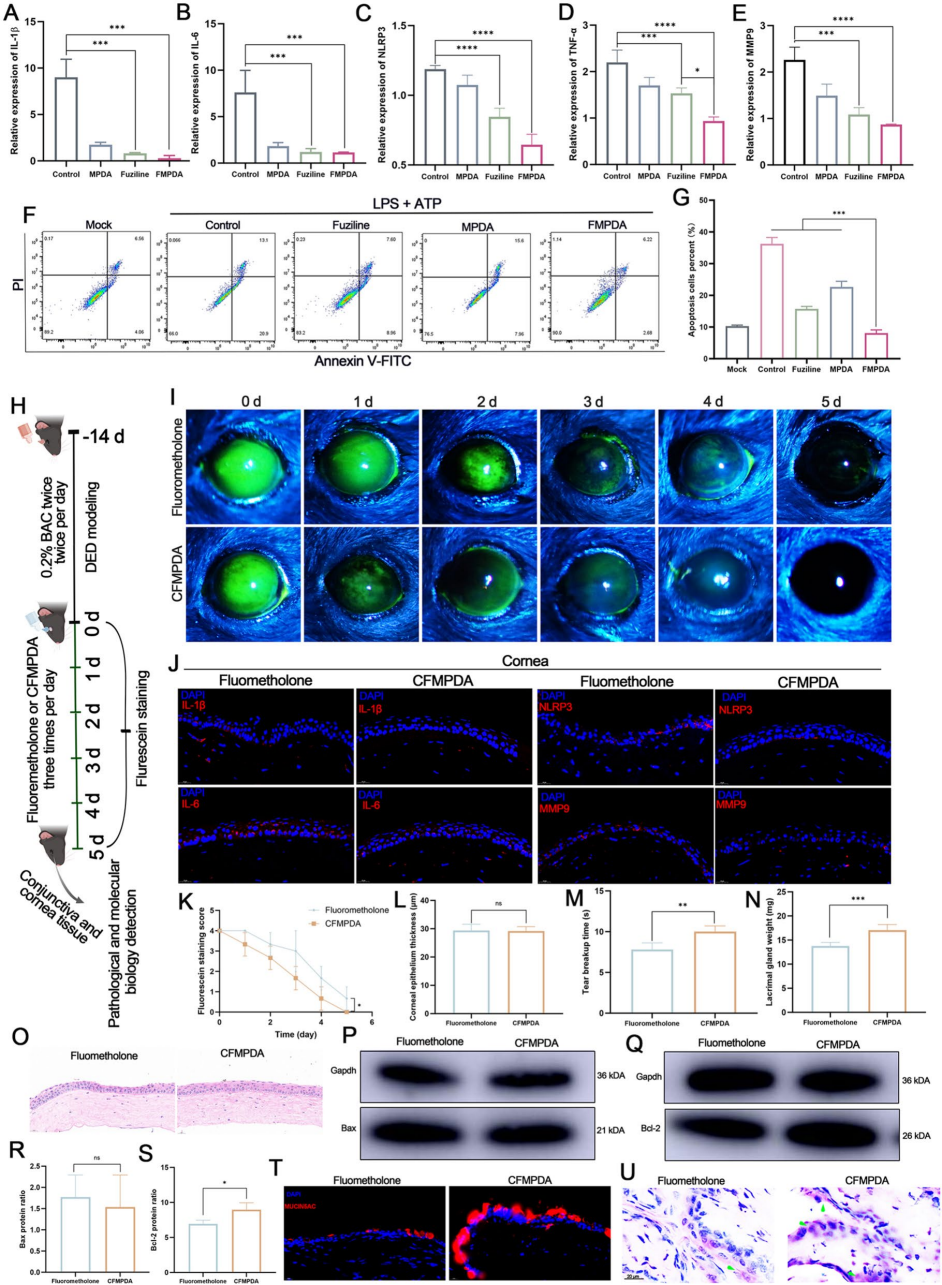
Therapeutic effects of nanoplatforms on the DED model. (**A**–**E**) mRNA levels of IL-1β, MMP-9, IL-6, TNF-α, and NLRP3 in HCECs after various treatments. (**F**) Flow cytometry analysis of apoptosis in HCECs after various treatments. (**G**) Quantitative analysis of flow cytometry data. (**H**) Schematic illustration of in vivo DED modeling and treatment. Therapeutic effects were evaluated and recorded daily during treatment. (**I**, **K**) Representative fluorescein sodium staining images and corresponding staining score analysis. (**J**) Representative immunofluorescence images of IL-1β, IL-6, NLRP3, and MMP9 in corneal sections. (**L**) Quantitative analysis of corneal epithelial thickness. (**M**) TBUT evaluation in mice. (**N**) Quantification of mouse lacrimal gland weight. (**O**) Representative H&E staining images of the cornea. (**P**, **R**) Western blot analysis of Bcl-2 expression in conjunctival tissue. (**Q**, **S**) Western blot analysis of Bax expression in conjunctival tissue. (**T**, **U**) Representative immunofluorescence images of MUCIN5AC and PAS staining in conjunctival sections. Data are presented as mean ± SD (n = 4). Significance was set at *P < 0.05, **P < 0.01, ***P < 0.001, ****P < *0.0001*, and ns = no significance. Statistical analyses were performed using two-tailed Student's t-test (**R**, **S**), one-way ANOVA (**A**–**E**, **G**, **L**, **M**, **N**), and two-way ANOVA (**K**)

The ultimate validation of a multifunctional delivery platform lies in its integrated therapeutic outcome. We evaluated the fully assembled CFMPDA nanoplatform in the DED mouse model, benchmarking it against the clinical standard, Fluorometholone, to assess whether the spatiotemporal coordination of targeting and dual therapeutic actions translates into superior efficacy and safety (Fig. 6H).

The nanoplatform demonstrated superior efficacy in resolving core ocular surface pathology. Daily fluorescein sodium staining revealed that CFMPDA treatment promoted faster and more complete corneal epithelial healing than Fluorometholone throughout the 5-day treatment course, culminating in a significantly lower final staining score (0 vs. 0.67, Fig. 6I and K). This was accompanied by a more robust restoration of tear film stability, as indicated by a longer tear break-up time (TBUT) in the CFMPDA group (10.0 ± 0.71 s vs. 7.8 ± 0.84 s, Fig. 6M). Histologically, both treatments restored corneal epithelium thickness to a similar degree (Fig. 6L and O), indicating comparable reparative capacity at the structural level.

A key differentiating advantage of CFMPDA was its ability to promote functional restoration of the lacrimal functional unit (LFU), a critical aspect often neglected by anti-inflammatory monotherapies. CFMPDA treatment resulted in significantly heavier lacrimal glands compared to the Fluorometholone group (17.08 ± 1.13 mg vs. 13.76 ± 0.75 mg, Fig. 6N), indicating a reversal of disease-associated atrophy. Furthermore, immunofluorescence and PAS staining of conjunctiva showed that CFMPDA markedly enhanced the density and mucin (MUCIN5AC) production of goblet cells (MFI: 20.37 vs. 4.05; area: 12.61% vs. 2.76%, Figs. 6T and U, S9F, G), which are essential for tear film integrity. This comprehensive restoration of the secretory apparatus highlights the benefit of concurrently addressing inflammation and mitochondrial oxidative stress (which directly damages secretory epithelia), a capability absent in corticosteroid monotherapy.

At the molecular level, CFMPDA effectively suppressed the inflammatory and oxidative pathways in vivo. PCR analysis in vitro had already shown that FMPDA (the core therapeutic combination) outperformed individual components in downregulating key mediators like IL-1β, IL-6, NLRP3, and TNF-α, and in reducing apoptosis (Fig. 6A).

A thorough biosafety assessment confirmed the excellent tolerability of the nanoplatform and its components. After 14 days of repeated ocular administration, mice treated with Fuziline, MPDA, or FMPDA showed no significant fluctuations in body weight or in key hematological and biochemical serum indices (Fig. S10A). Most importantly, histopathological examination (H&E staining) of major organs (heart, liver, spleen, lungs, kidneys) revealed no detectable lesions or signs of toxicity compared to control animals (Fig. S10B). This compelling safety profile underscores the biocompatibility of the polydopamine-based nanomaterial and supports the potential for clinical translation.

In conclusion, the CFMPDA nanoplatform delivers on its engineered promise: it integrates precision targeting at two hierarchical levels (diseased tissue via CCL2 and damaged organelle via mitochondrial tropism) with spatiotemporally coordinated dual therapy (anti-inflammation and anti-oxidant). This results in comprehensive and superior therapeutic outcomes that not only match the anti-inflammatory potency of a standard steroid but surpass it by functionally restoring the lacrimal apparatus. This work validates a novel nanotherapeutic strategy where intelligent delivery system design is leveraged to achieve synergistic pharmacology against a complex multifactorial disease.

## Conclusion

In conclusion, this work demonstrates the successful engineering and validation of a hierarchically functional nanoplatform (CFMPDA), which operationalizes a rational “sense-and-actuate” strategy to actively navigate the multiscale barriers inherent in ocular drug delivery. This platform is architected by integrating three distinct, complementary modules into a single nanoconstruct: (1) a mesoporous polydopamine (MPDA) core serving as both an intrinsic reactive oxygen species (ROS)-scavenging nanoscavenger and a drug reservoir; (2) a broad-spectrum anti-inflammatory payload (Fuziline); and (3) surface-conjugated CCL2-targeting antibodies for active recognition.

The programmed intelligence of this material system is validated through its spatiotemporally orchestrated functions. First, it achieves pathology-responsive bio-interfacing: the surface antibodies enable active recognition and binding to the CCL2-rich inflamed ocular surface, fundamentally altering its pharmacokinetics from passive clearance to disease severity-dependent retention, thereby solving the primary challenge of rapid precorneal drug loss. Subsequently, it executes programmed subcellular trafficking: leveraging the inherent mitochondrial tropism of the MPDA core, the system localizes precisely to the organelle responsible for oxidative damage, ensuring therapeutic action is delivered at the pathological source.

This rational material design translated directly into superior therapeutic efficacy. In a murine model of dry eye disease (DED), CFMPDA outperformed the clinical standard (Fluorometholone) by not only effectively suppressing corneal inflammation but also uniquely promoting functional restoration of the lacrimal functional unit. This holistic outcome underscores the critical advantage of a material strategy that concurrently disrupts both inflammatory signaling and mitochondrial oxidative stress—a synergy unattainable by single-mechanism therapies. The excellent biosafety profile of all components further supports the translational promise of this polydopamine-based platform.

Beyond presenting a potent nanotherapeutic for DED, this study establishes a generalizable materials-centric blueprint. The core design principle—coupling biomarker-responsive tissue anchoring with programmed organelle-specific delivery—creates a versatile framework for engineering "active" therapeutic materials. This strategy is readily adaptable to a wide spectrum of localized inflammatory pathologies (e.g., rheumatoid arthritis, dermatitis, colitis) where defined chemokine upregulation and mitochondrial dysfunction coexist. Therefore, our work advances the frontier of therapeutic biomaterials by showcasing how precise nano-engineering can create intelligent systems that actively navigate biological complexity to achieve synergistic and tissue-restorative therapy.

## Supplementary Information

Supplementary Material 1.

Supplementary Material 2 : **Fig. S1** (**A**) Chord diagram showing GO enrichment analysis of the top 11 hub cytokines. (**B**) Concentrations of 64 cytokines in the supernatant of normal HCECs and LPS+ATP-induced HCECs. (**C**) STRING protein–protein interaction network of cytokines with a confidence score ≥1000. (**D**) Heatmap showing interactions among the top 14 hub cytokines. (**E**) Chord diagram showing GO enrichment analysis of the top 14 hub cytokines.

Supplementary Material 3 : **Fig. S2** (**A**) Representative fluorescein sodium staining images and the corresponding staining score analysis. (**D**) Representative immunofluorescence images of CD3, CD4 and IL-23R in corneal sections and MFI quantification of immunofluorescence signal. (**G**) Representative immunofluorescence images of monocyte surface antigen in corneal sections. (**B**, **C**, **E**, **F**, **H**, **I** and **J**) RNA expression levels of immune-related genes in the control and CCL2 antibody groups. Data are presented as mean ± SD (n = 3). Significance was set at *P < 0.05, **P < 0.01, ***P < 0.001, ****P < 0.0001, and ns = no significance. Statistical analyses in (**A**, **B**, **C**, **D**, **E**, **F**, **H**, **I** and **J**) were performed using a two-tailed Student's t-test.

Supplementary Material 4: **Fig. S3** (**A**) CCK-8 assay determining the optimal therapeutic concentration of Fuziline in LPS+ATP-induced HCECs. (**B**) RNA levels of CCL2 in LPS+ATP-induced HCECs after treatment with fuziline. (**C**) Heatmap showing RNA expression levels of immune-related genes in the control and fuziline groups. (**D**, **E**) Representative fluorescein sodium staining images and the corresponding staining score analysis. All data are presented as mean ± SD (n = 3). Significance was set at *P < 0.05, **P < 0.01, ***P < 0.001, and ****P < 0.0001. Statistical analyses were performed using one-way ANOVA (**A**) and two-tailed Student's t-test (**B**, **C**)

Supplementary Material 5: **Fig. S4** (**A**, **B**) Representative fluorescein sodium staining images and the corresponding staining score analysis. (**C**) MFI quantification of IL-1β and ROS immunofluorescence signals in corneal sections of the control and MPDA groups. (**D**) MFI quantification of IL-1β and ROS immunofluorescence signals in conjunctival sections of the control and MPDA groups. (**E**) TUNEL staining of corneal sections from the control and MPDA groups. (**F**) Quantification of TUNEL-positive cells in corneal sections. (**G**) TUNEL staining of conjunctival sections from the control and MPDA groups. (**H**) Quantification of TUNEL-positive cells in conjunctival sections. All data are presented as mean ± SD (n = 3). Significance was set at *P < 0.05, **P < 0.01, ***P < 0.001, and ****P < 0.0001. Statistical analyses in (**B**–**D**, **F**, **H**) were performed using a two-tailed Student's t-test

Supplementary Material 6 : **Fig. S5 **(**A**) Schematic illustration of PDA formation from DA. (**B**) TEM images of MPDA and FMPDA. (**C**) UV–vis absorbance spectra of Fuziline, MPDA, FMPDA, and CFMPDA. (**D**) Fourier transform infrared spectroscopy of Fuziline, MPDA, FMPDA, and CFMPDA. (**E**) Calibration curve of Fuziline for quantification of its loading efficiency using UPLC-MS/MS. (**F**) In vitro release profile of Fuziline from FMPDA and CFMPDA. (**G**) CCK-8 assay determining the optimal therapeutic concentration of MPDA in XOD-induced HCECs. All data are presented as mean ± SD (n = 3). Significance was set at *P < 0.05, **P < 0.01, ***P < 0.001, and ****P < 0.0001. Linear regression was performed in (**E**), and one-way ANOVA was used in (**F**)

Supplementary Material 7 : **Fig. S6 **(**A**) Representative CLSM images of HCECs stained with JC-1 after different treatments and the corresponding quantification of the red/green fluorescence ratio. (**B**) Representative CLSM images of HCECs stained with PI and calcein after different treatments and the corresponding quantification of live/dead cells. All data are presented as mean ± SD (n = 3). Significance was set at *P < 0.05, **P < 0.01, ***P < 0.001, and ****P < 0.0001. Statistical analyses in (**A**) and (**B**) were performed using one-way ANOVA

Supplementary Material 8: **Fig. S7 **(**A**) Representative images of fluorescence signals from CY5-SE-labeled FMPDA at different concentrations in vitro (10 μL of nanoplatform suspension per well). (**B**) Correlation between fluorescence intensity and concentration of CY5-SE-labeled FMPDA. (**C**) Representative images of fluorescence signals from CY5-SE-labeled CFMPDA at different concentrations in vitro (10 μL of nanoplatform suspension per well). (**D**) Correlation between fluorescence intensity and concentration of CY5-SE-labeled CFMPDA. (**E**) Representative ex vivo fluorescence images of eyeballs dissected 60 min after administration of CY5-SE-labeled nanoplatforms. (**F**) Standard curve of fluorescence intensity versus concentration for CY5-SE-labeled FMPDA and CFMPDA (10 μL of nanoplatform suspension per well). (**G**) Concentration profile of Fuziline in the cornea at different time points after eye drop administration (Fuziline: 300 μg/mL, 5 μL). (**H**, **I**, **J**) Quantitative analysis of IVIS fluorescence signals. All data are presented as mean ± SD (n = 3). Significance was set at *P < 0.05, **P < 0.01, ***P < 0.001, and ****P < 0.0001. Linear regression was performed in (B) and (D), and one-way ANOVA was used in (**F**, **H**, **I**, **J**)

Supplementary Material 9 : **Fig. S8** (**A**) Flow cytometry analysis of intracellular uptake of CY5-SE-labeled MPDA (300 μg/mL) by HCECs after LPS induction for various times. (**B**) Flow cytometry analysis of intracellular uptake of CY5-SE-labeled FMPDA (300 μg/mL) by HCECs after LPS induction for various times. (**C**) Quantification of flow cytometry results from (**A**) and (**B**). (**D**) Percentage of MitoSOX-positive HCECs. (**E**) Quantitative analysis of Pearson correlation coefficients for MPDA, FMPDA, and CFMPDA. All data are presented as mean ± SD (n = 3). Significance was set at *P < 0.05, **P < 0.01, ***P < 0.001, ****P < 0.0001, and ns = no significance. Statistical analyses in (**D**) and (**E**) were performed using one-way ANOVA

Supplementary Material 10: **Fig. S9** (**A**) CCK-8 assay demonstrating the optimal therapeutic concentration of FMPDA in LPS+ATP-induced HCECs. (**B**–**E**) MFI quantification of IL-1β, IL-6, NLRP3, and MMP-9 immunofluorescence signals in corneal sections. (**F**) MFI quantification of MUCIN5AC immunofluorescence signals in conjunctival sections. (**G**) Quantification of goblet cell area. All data are presented as mean ± SD (n = 4). Significance was set at *P < 0.05, **P < 0.01, ***P < 0.001, ****P < 0.0001, and ns = no significance. Statistical analyses were performed using one-way ANOVA (**A**) and two-tailed Student's t-test (**B**–**G**)

Supplementary Material 11: **Fig. S10** (**A**) Body weight and blood analysis of mice after various treatments for 14 days (n = 5). (**B**) H&E staining of histological sections of heart, liver, spleen, lung, and kidney samples collected from mice after various treatments for 14 days. All data are presented as mean ± SD (n = 5). Significance was set at *P < 0.05, **P < 0.01, ***P < 0.001, ****P < 0.0001, and ns = no significance. Statistical analysis in (**A**) was performed using one-way ANOVA

## Data Availability

The datasets used and/or analysed during the current study are available from the corresponding author on reasonable request. No datasets were generated or analysed during the current study.
