# Supplementary material for: A sequentially targeted and pathology-responsive nanoplatform for synergistic treatment of dry eye disease via concurrent anti-inflammation and mitochondrial ROS scavenging
Source: J Nanobiotechnology. 2026 May 22;24:674. doi: 10.1186/s12951-026-04365-7 (PMC13383135; doi:10.1186/s12951-026-04365-7)
Supplement: Supplementary file 1 — Supplementary Material 1. [file 12951_2026_4365_MOESM1_ESM.docx]

**A Sequentially Targeted and Pathology-Responsive Nanoplatform for Synergistic Treatment of Dry Eye Disease via Concurrent Anti-Inflammation and Mitochondrial ROS Scavenging**

Liandi Huang ^1, 2^, Xin Liu ^3^, Ying Yuan ^1, 2^, Bilian Ke ^1, 3^

1. Department of Ophthalmology, Shanghai General Hospital, Shanghai Jiao Tong University School of Medicine, No. 100 Haining Road, Shanghai, 200080, China
2. National Clinical Research Center for Eye Diseases, Shanghai Key Laboratory of Ocular Fundus Diseases, Shanghai Engineering Center for Visual Science and Photomedicine, Shanghai Engineering Center for Precise Diagnosis and Treatment of Eye Diseases, Shanghai, PR China
3. Department of Ophthalmology, Shanghai Renji Hospital, Shanghai Jiao Tong University School of Medicine, No. 160 Pujian Road, Shanghai, 200127, China

Corresponding author:

Ke Bilian, Prof

Department of Ophthalmology, Shanghai Renji Hospital, Shanghai Jiao Tong University School of Medicine, No. 160 Pujian Road, Shanghai, 200127, China

E-mail adress: kebilian@sjtu.edu.cn

**Supplementary methods**

**Cells**

Human monocytic leukemia cells (THP-1) and human corneal epithelial cells (HCECs) were obtained from the American Type Culture Collection (ATCC). HCECs were cultured in SFM (1X) medium, while THP-1 cells were cultured in RPMI 1640 medium. Both media were supplemented with 10% fetal bovine serum (FBS) and 1% penicillin-streptomycin. All cells were maintained at 37 °C in a humidified incubator with 5% CO_2_.

**CCk-8 assay**

**Determination of Optimal Therapeutic Concentrations of Fuziline, MPDA, and FMPDA Using the CCK-8 Assay**

**Optimal anti-inflammatory concentration of Fuziline:** HCECs were seeded into 96-well plates at a density of 5.0 × 10³ cells/well and cultured for 24 h. The cells were then divided into three groups: mock, LPS+ATP, and treatment. Cells in the LPS+ATP and treatment groups were sequentially exposed to LPS (1 μg/mL) for 12 h and ATP (5 mM) for 1 h. After induction, the cells were washed three times with PBS. Subsequently, 100 μL of basal medium was added to the LPS+ATP group, while the treatment group received 100 μL of basal medium containing Fuziline at concentrations of 10, 20, 30, 40, or 50 μg/mL. Following 48 h of incubation, the cells were washed three times with PBS, and 100 μL of 10% CCK-8 solution was added to each well. After 30 min of incubation, the absorbance at 450 nm was measured using a microplate reader. The optimal anti-inflammatory concentration of Fuziline was determined by comparison with the LPS+ATP group.

**Optimal antioxidant concentration of MPDA:** HCECs were seeded into 96-well plates at a density of 5.0 × 10³ cells/well and cultured for 24 h. The cells were then divided into three groups: mock, XOD, and treatment. Cells in the XOD and treatment groups were incubated with XOD (70 U/L) for 24 h. After induction, the cells were washed three times with PBS. Subsequently, 100 μL of basal medium was added to the XOD group, while the treatment group received 100 μL of basal medium containing MPDA at concentrations of 50, 60, 70, 80, 90, or 100 μg/mL. Following 48 h of incubation, the cells were washed three times with PBS, and 100 μL of 10% CCK-8 solution was added to each well. After 30 min of incubation, the absorbance at 450 nm was measured using a microplate reader. The optimal antioxidant concentration of MPDA was determined by comparison with the XOD group.

**Optimal concentrations of FMPDA:** The optimal anti-inflammatory and antioxidant concentrations of FMPDA were determined *in vitro* following the same procedures described above.

**Verification of Fuziline-Mediated CCL2 Regulation in HCECs and THP-1 Cells**

To verify the effect of Fuziline on CCL2 expression, HCECs and THP-1 cells (3 × 10⁵ cells/well) were seeded separately in six-well plates and cultured for 12 h. All cells were then sequentially stimulated with LPS (1 μg/mL) for 12 h and ATP (5 mM) for 1 h. After induction, the cells were divided into two groups: a control group receiving basal medium and a Fuziline group receiving basal medium containing Fuziline (40 μg/mL). Following 24 h of incubation, cells were harvested for quantitative real-time PCR (qRT-PCR) analysis.

Total RNA was extracted from HCECs and THP-1 cells using TRIzol reagent (Life Technologies, CA, USA). After quantification, RNA was reverse transcribed into cDNA using the Takara Reverse Transcription Kit (Takara, Japan). The cDNA products were subjected to qRT-PCR on an Applied Biosystems instrument (ABI, Foster City, CA, USA). Relative gene expression was calculated using the 2⁻ΔΔCt method, with GAPDH serving as the internal control.

**Comparison of Anti-Inflammatory Effects of Fuziline, MPDA, and FMPDA**

To compare the anti-inflammatory effects of Fuziline, MPDA, and FMPDA, HCECs (3 × 10⁵ cells/well) were seeded in six-well plates and cultured for 12 h. All cells were sequentially stimulated with LPS (1 μg/mL) for 12 h and ATP (5 mM) for 1 h. After induction, the cells were divided into four groups: a control group receiving basal medium, and treatment groups receiving Fuziline (40 μg/mL), MPDA (40 μg/mL), or FMPDA (40 μg/mL). After 24 h of incubation, cells were harvested, and qRT-PCR was performed following the procedures described above.

The sequences of the primers used in this study were in Table 1

Table 1. Primers sequences used for qRT-PCR

| Name | Forward | Reverse |
| --- | --- | --- |
| CCL-2 | 5′-CAGCCAGATGCAATCAATGCC- 3′ | 5′-TGGAATCCTGAACCCACTTCT- 3′ |
| IL-1β | 5′-ATGATGGCTTATTACAGTGGCAA- 3′ | 5′-GTCGGAGATTCGTAGCTGGA- 3′ |
| NLRP3 | 5′-CGTGAGTCCCATTAAGATGGAGT- 3′ | 5′-CCCGACAGTGGATATAGAACAGA- 3′ |
| IL-6 | 5′-ACTCACCTCTTCAGAACGAATTG- 3′ | 5′-CCATCTTTGGAAGGTTCAGGTTG- 3′ |
| MMP9 | 5′-AGAGATGCGTGGAGAGTCG- 3 | 5′-AGTCTTCCGAGTAGTTTTGG- 3 |
| GAPDH | 5′-GGAGCGAGATCCCTCCAAAAT- 3′ | 5′-GGCTGTTGTCATACTTCTCATGG- 3′ |
| TNF-α | 5′-GGAGCGAGATCCCTCCAAAAT- 3′ | 5′-CGGGCCGATTGATCTCAGC- 3′ |

**Cellular Uptake of MPDA and FMPDA**

The cellular uptake of MPDA and FMPDA by HCECs was evaluated using flow cytometry. HCECs were seeded into six-well plates at a density of 5 × 10³ cells/well and cultured for 12 h. The culture medium was then replaced with fresh medium containing LPS (1 μg/mL). After LPS treatment for various durations (2, 4, 8, 12, and 24 h), the medium was discarded, and the cells were washed twice with PBS. Subsequently, fresh medium containing CY5-SE-labeled MPDA or FMPDA (100 μg/mL) was added to the corresponding wells. Following 4 h of incubation, all cells were collected, and the percentage of CY5-positive cells was analyzed by flow cytometry. The cellular uptake of FMPDA was assessed following the same procedure.

**Verification of therapeutic effects of CCL2 antibody, Fuziline and MDPA on DED**

After successful establishment of the DED mouse model, the animals were divided into two groups: a control group treated with saline and a CCL2 antibody group treated with CCL2 antibody (10 μL, 500 μg/mL) three times daily. After 5 days of treatment, all animals were anesthetized via intraperitoneal injection of sodium pentobarbital (200 mg/kg). Corneas were then stained with 1 μL of 1% fluorescein sodium and observed under a slit-lamp microscope. Corneal fluorescein staining was quantified using a scoring system ranging from 0 to 4 (0 = no staining, 1 = diffuse dot-like green staining, 2 = green-staining area less than one-third of the cornea, 3 = green-staining area greater than one-third of the cornea, and 4 = green-staining area over two-thirds of the cornea) [1].

Fuziline (10 μL, 300 μg/mL) and MPDA (10 μL, 800 μg/mL) were used to treat DED mice following the same experimental protocol described above.

**Pharmacokinetic Study of Fuziline**

After being fixed to expose the eyeballs, the mice were administered 5 μL of 2% Fuziline eye drops into the conjunctival sac of both eyes. The eyelids were gently closed for 1 minute. Following a single administration, the mice were sacrificed with CO₂ at 5, 15, 30, 45, 60, 90, 120, 150, and 180 minutes. The eyeballs were thoroughly rinsed with saline, and the corneas were extracted, with non-corneal tissues being removed. Each time point was repeated 3 times, and the number of corneas collected was as follows: 2, 2, 4, 6, 8, 8, 10, 10, and 12 per time point, respectively. The Fuziline concentrations in the cornea were measured using UPLC-MS/MS. The concentration–time curve was determined using Phoenix WinNonlin 8.3 software (Certara, Saint Louis, MO, USA). Then Thirty DED mice were divided into three treatment groups: Fuziline, FMPDA, and CFMPDA. Each group received 5 μL of Fuziline (300 μg/mL), FMPDA (300 μg/mL), or CFMPDA (300 μg/mL) eye drops. After 60 minutes, the mice were sacrificed, and the corneas were dissected. Fuziline concentrations in the corneas were measured using UPLC-MS/MS.

**In Vitro Drug Release Study of Fuziline from FMPDA and CFMPDA**

To evaluate the *in vitro* release profile of Fuziline from the nanoplatforms, 5 mg each of FMPDA and CFMPDA were separately dispersed in 5 mL of phosphate-buffered saline (PBS) and incubated at 37 °C with gentle shaking at 40 rpm. At predetermined time intervals (2, 4, 8, 12, 24, 48, and 72 h), 50 μL aliquots of the suspension were collected and immediately centrifuged at 10,000 rpm to pellet the nanoparticles. The supernatant was carefully collected, and the concentration of released Fuziline was quantified using ultra-performance liquid chromatography-tandem mass spectrometry (UPLC-MS/MS). The cumulative drug release percentage was calculated based on the initial Fuziline loading amount. All experiments were performed in triplicate, and the results are presented as mean ± SD

**Cytokine multiplex assay and bioinformatic analysis in vitro**

The Invitrogen™ ProcartaPlex™ Human 65-plex panel (EPX650-10065-901, ThermoFisher Scientific, Waltham, MA, USA) was used to detect cytokine levels in the supernatant of normal and inflamed cells. Briefly, supernatant samples were centrifuged at 10,000×g for 10 min. The samples and a dilution series of the lyophilized standard mix (containing 65 proteins) were loaded onto the plate and incubated. After washing, the beads were incubated with primary antibodies, washed, incubated with secondary antibodies, washed again, and finally resuspended in reading solution. Plates were analyzed using a Luminex 200 System [2].

For data analysis, cytokine interaction networks were constructed using the STRING database (confidence score ≥ 1000). The top 14 hub cytokines were identified by degree centrality and subsequently subjected to: (1) Ingenuity Pathway Analysis (IPA) to predict functional interactions and regulatory pathways, and (2) KEGG pathway enrichment analysis to map hub cytokines to biologically relevant pathways.

**In Vivo Biocompatibility Studies**

Twenty-five mice were randomly assigned to five groups and received topical instillation into the left eye three times daily for 14 consecutive days. The treatments administered were: 10 µL of 0.9% saline (w/v) for the control group, and 10 µL of fuziline (300 μg/mL), MPDA (800 μg/mL), FMPDA (300 μg/mL), or CFMPDA (300 μg/mL) for the respective treatment groups. On day 14, body weights were recorded, and blood samples were collected for hematological and biochemical analysis. Following euthanasia by anesthetic overdose, major organs (liver, lung, heart, kidney, and spleen) were promptly harvested and processed into H&E-stained sections for biosafety assessment.

**References:**

1. Shao M, Chai Y, Jiang Y, Wu X, Xie W, Lu J, Fu X, He Y, Zhang X, Zhang H, Liu Z: **Eye-Drop Nano-Formulation of Catalase Self-Assembled with Thiolated Chitosan for Effective Treatment of Dry Eye Disease.** *Adv Mater* 2025, **37:**e2415353.

2. Cook DB, McLucas BC, Montoya LA, Brotski CM, Das S, Miholits M, Sebata TH: **Multiplexing protein and gene level measurements on a single Luminex platform.** *Methods* 2019, **158:**27-32.
