# Supplementary figures and images for: A sequentially targeted and pathology-responsive nanoplatform for synergistic treatment of dry eye disease via concurrent anti-inflammation and mitochondrial ROS scavenging
Source: J Nanobiotechnology. 2026 May 22;24:674. doi: 10.1186/s12951-026-04365-7 (PMC13383135; doi:10.1186/s12951-026-04365-7)

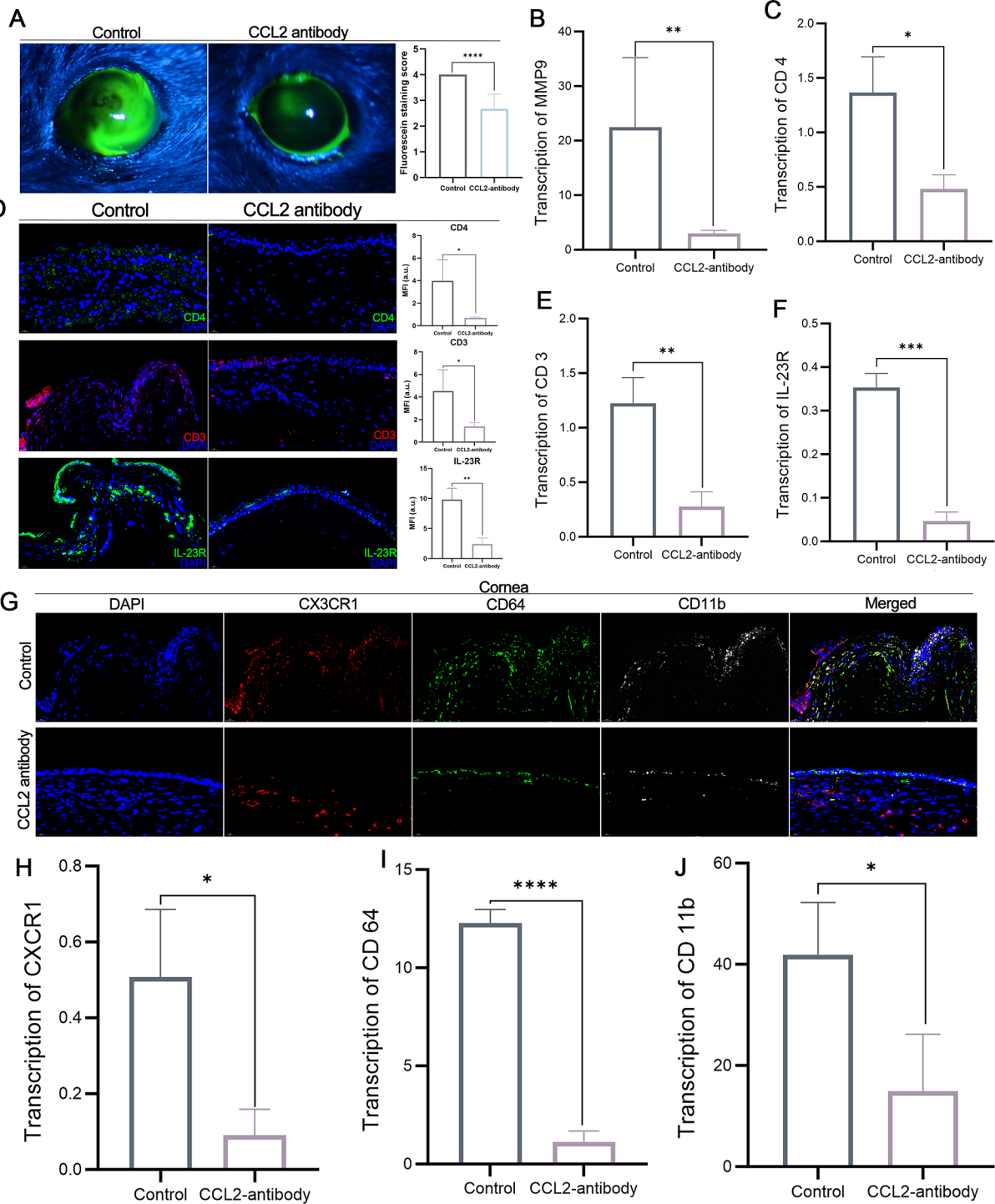

Supplement: Supplementary file 3 — Supplementary Material 3 : Fig. S2 (A) Representative fluorescein sodium staining images and the corresponding staining score analysis. (D) Representative immunofluorescence images of CD3, CD4 and IL-23R in corneal sections and MFI quantification of immunofluorescence signal. (G) Representative immunofluorescence images of monocyte surface antigen in corneal sections. (B, C, E, F, H, I and J) RNA expression levels of immune-related genes in the control and CCL2 antibody groups. Data are presented as mean ± SD (n = 3). Significance was set at *P < 0.05, **P < 0.01, ***P < 0.001, ****P < 0.0001, and ns = no significance. Statistical analyses in (A, B, C, D, E, F, H, I and J) were performed using a two-tailed Student's t-test. [file 12951_2026_4365_MOESM3_ESM.pdf]

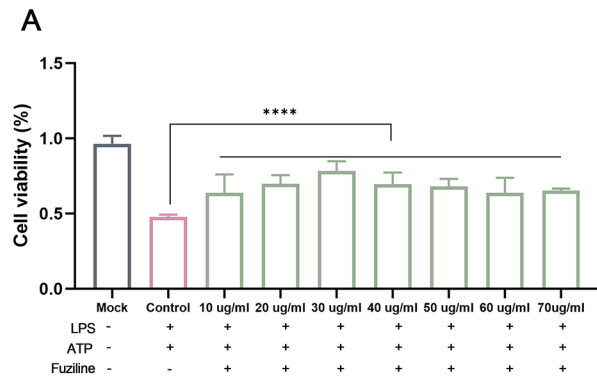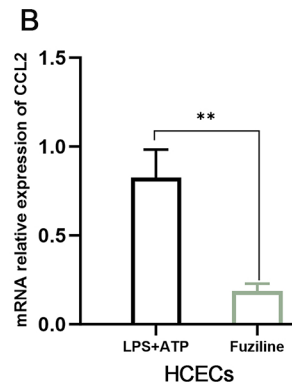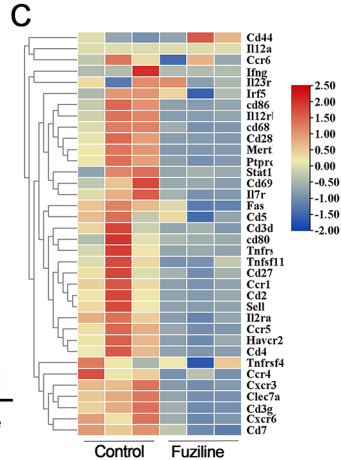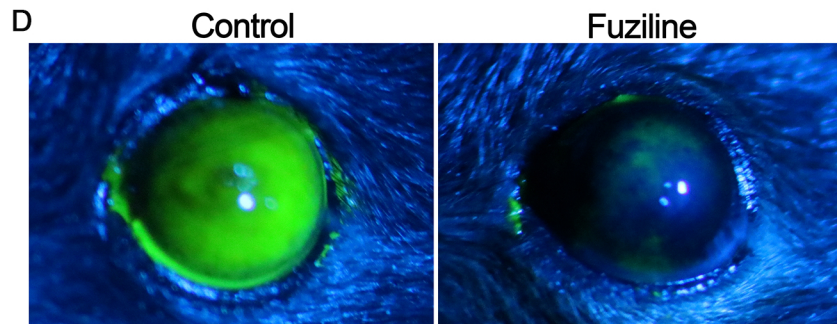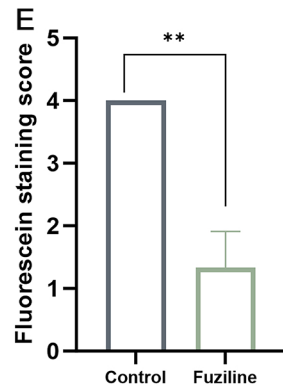

Supplement: Supplementary file 4 — Supplementary Material 4: Fig. S3 (A) CCK-8 assay determining the optimal therapeutic concentration of Fuziline in LPS+ATP-induced HCECs. (B) RNA levels of CCL2 in LPS+ATP-induced HCECs after treatment with fuziline. (C) Heatmap showing RNA expression levels of immune-related genes in the control and fuziline groups. (D, E) Representative fluorescein sodium staining images and the corresponding staining score analysis. All data are presented as mean ± SD (n = 3). Significance was set at *P < 0.05, **P < 0.01, ***P < 0.001, and ****P < 0.0001. Statistical analyses were performed using one-way ANOVA (A) and two-tailed Student's t-test (B, C) [file 12951_2026_4365_MOESM4_ESM.pdf]

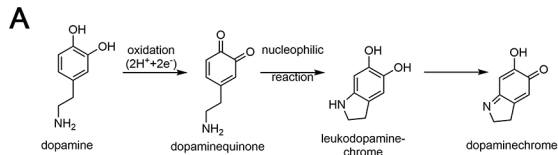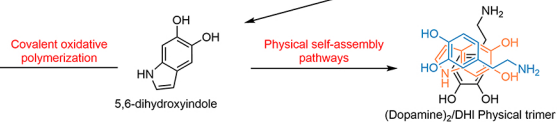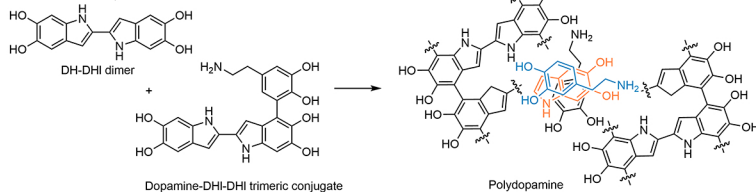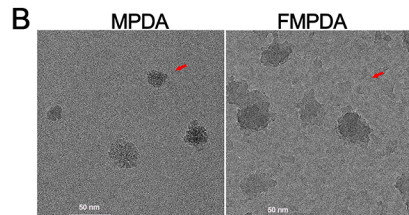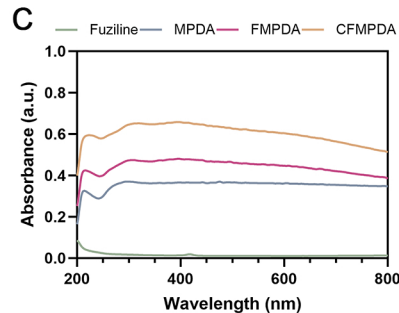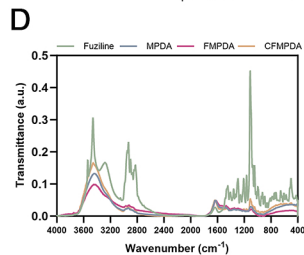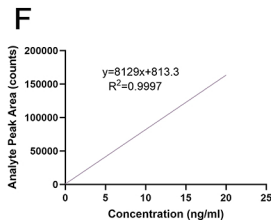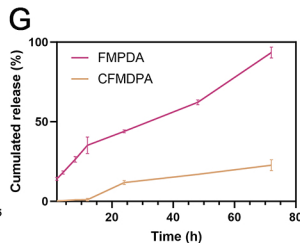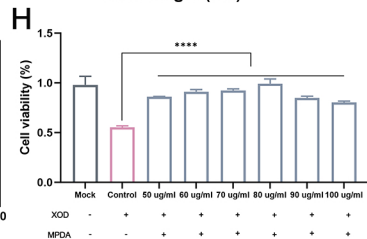

Supplement: Supplementary file 6 — Supplementary Material 6 : Fig. S5 (A) Schematic illustration of PDA formation from DA. (B) TEM images of MPDA and FMPDA. (C) UV–vis absorbance spectra of Fuziline, MPDA, FMPDA, and CFMPDA. (D) Fourier transform infrared spectroscopy of Fuziline, MPDA, FMPDA, and CFMPDA. (E) Calibration curve of Fuziline for quantification of its loading efficiency using UPLC-MS/MS. (F) In vitro release profile of Fuziline from FMPDA and CFMPDA. (G) CCK-8 assay determining the optimal therapeutic concentration of MPDA in XOD-induced HCECs. All data are presented as mean ± SD (n = 3). Significance was set at *P < 0.05, **P < 0.01, ***P < 0.001, and ****P < 0.0001. Linear regression was performed in (E), and one-way ANOVA was used in (F) [file 12951_2026_4365_MOESM6_ESM.pdf]

A

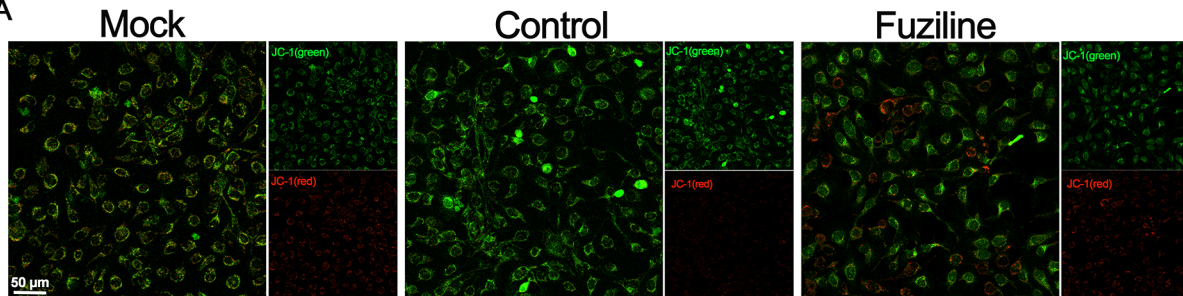

MPDA

FMPDA

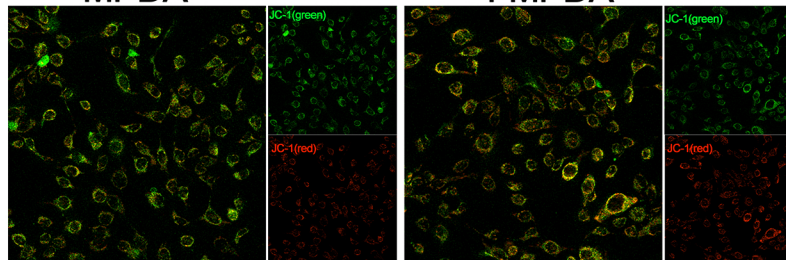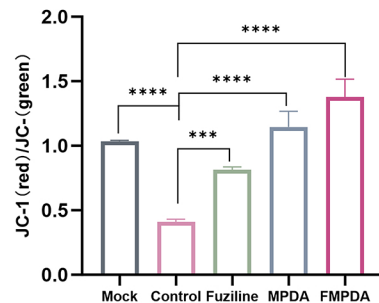

B

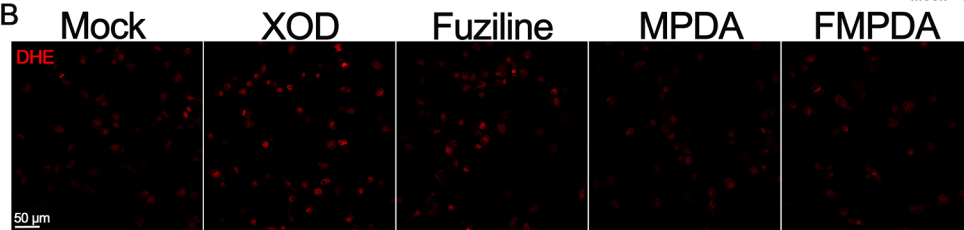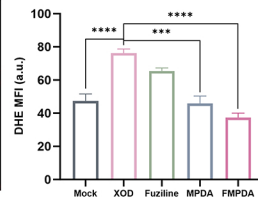

Supplement: Supplementary file 7 — Supplementary Material 7 : Fig. S6 (A) Representative CLSM images of HCECs stained with JC-1 after different treatments and the corresponding quantification of the red/green fluorescence ratio. (B) Representative CLSM images of HCECs stained with PI and calcein after different treatments and the corresponding quantification of live/dead cells. All data are presented as mean ± SD (n = 3). Significance was set at *P < 0.05, **P < 0.01, ***P < 0.001, and ****P < 0.0001. Statistical analyses in (A) and (B) were performed using one-way ANOVA [file 12951_2026_4365_MOESM7_ESM.pdf]

A

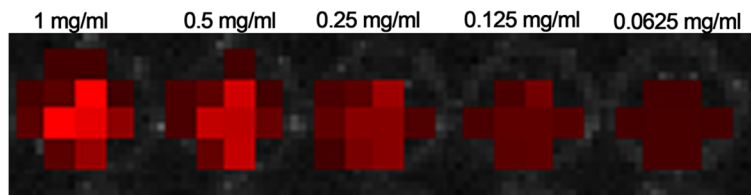

B

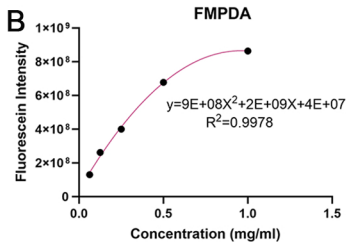

C

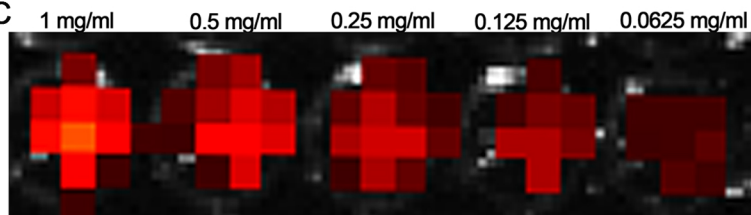

D

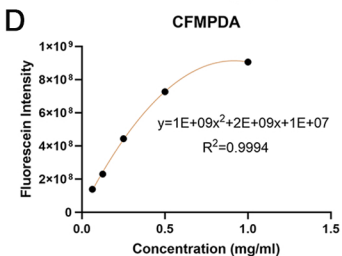

E

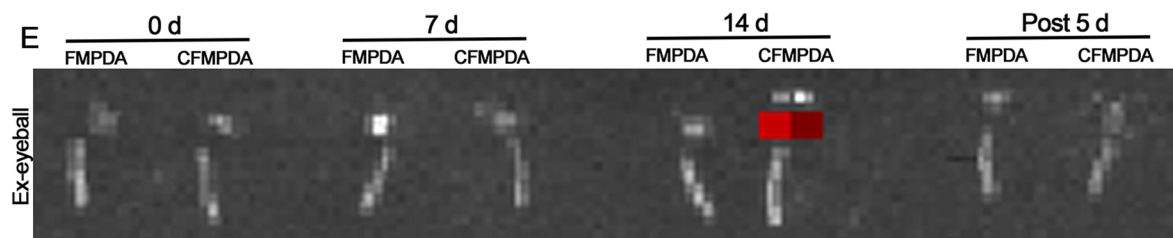

F

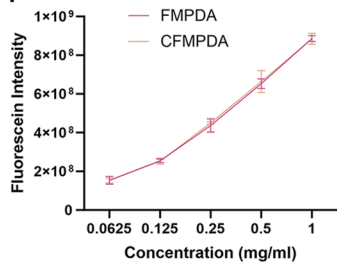

G

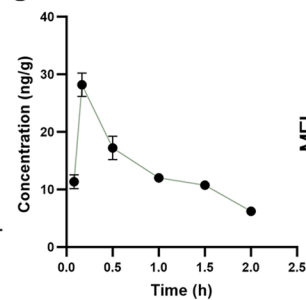

H

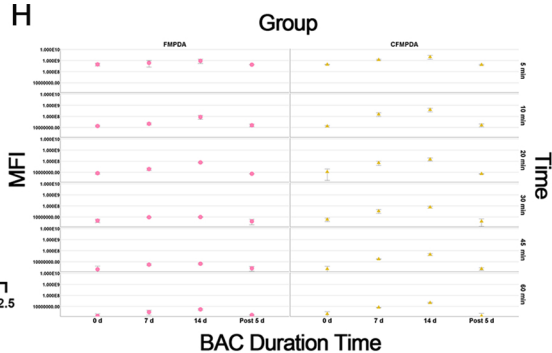

I

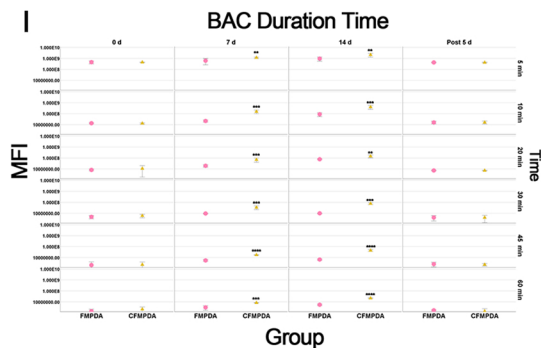

J

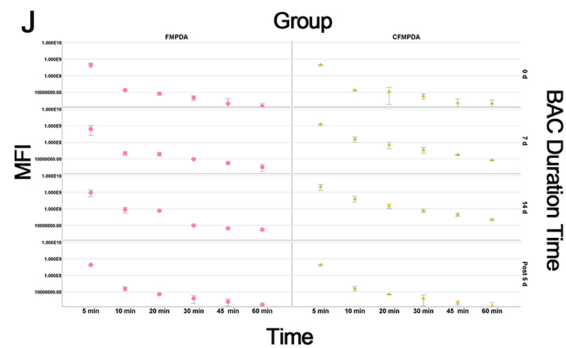

Supplement: Supplementary file 8 — Supplementary Material 8: Fig. S7 (A) Representative images of fluorescence signals from CY5-SE-labeled FMPDA at different concentrations in vitro (10 μL of nanoplatform suspension per well). (B) Correlation between fluorescence intensity and concentration of CY5-SE-labeled FMPDA. (C) Representative images of fluorescence signals from CY5-SE-labeled CFMPDA at different concentrations in vitro (10 μL of nanoplatform suspension per well). (D) Correlation between fluorescence intensity and concentration of CY5-SE-labeled CFMPDA. (E) Representative ex vivo fluorescence images of eyeballs dissected 60 min after administration of CY5-SE-labeled nanoplatforms. (F) Standard curve of fluorescence intensity versus concentration for CY5-SE-labeled FMPDA and CFMPDA (10 μL of nanoplatform suspension per well). (G) Concentration profile of Fuziline in the cornea at different time points after eye drop administration (Fuziline: 300 μg/mL, 5 μL). (H, I, J) Quantitative analysis of IVIS fluorescence signals. All data are presented as mean ± SD (n = 3). Significance was set at *P < 0.05, **P < 0.01, ***P < 0.001, and ****P < 0.0001. Linear regression was performed in (B) and (D), and one-way ANOVA was used in (F, H, I, J) [file 12951_2026_4365_MOESM8_ESM.pdf]

**A****MPDA**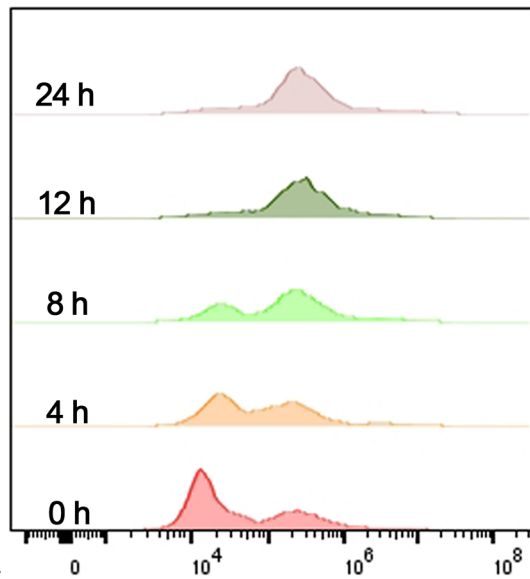**B****FMPDA**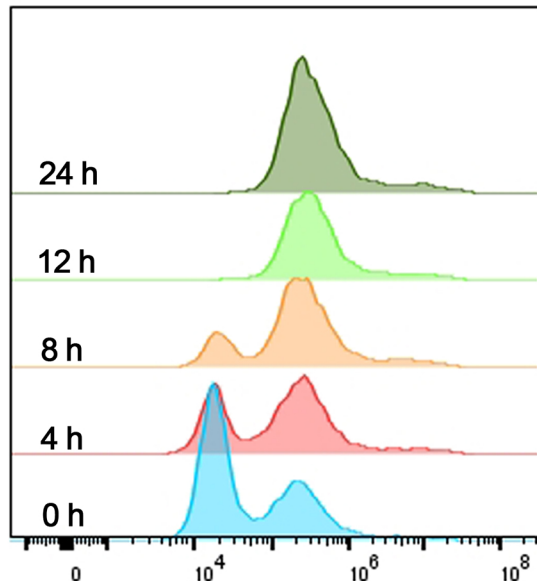**C**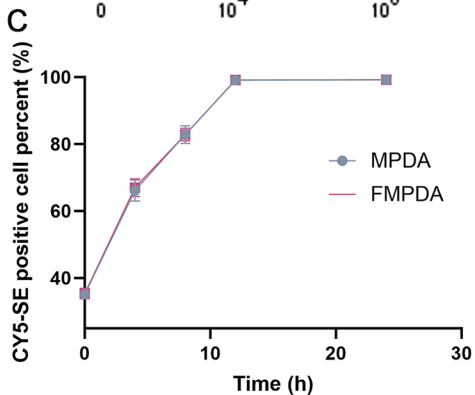**D**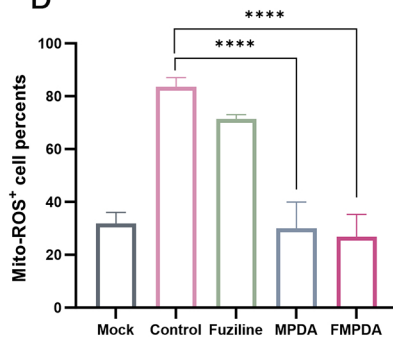**E**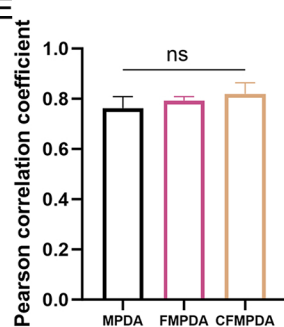

Supplement: Supplementary file 9 — Supplementary Material 9 : Fig. S8 (A) Flow cytometry analysis of intracellular uptake of CY5-SE-labeled MPDA (300 μg/mL) by HCECs after LPS induction for various times. (B) Flow cytometry analysis of intracellular uptake of CY5-SE-labeled FMPDA (300 μg/mL) by HCECs after LPS induction for various times. (C) Quantification of flow cytometry results from (A) and (B). (D) Percentage of MitoSOX-positive HCECs. (E) Quantitative analysis of Pearson correlation coefficients for MPDA, FMPDA, and CFMPDA. All data are presented as mean ± SD (n = 3). Significance was set at *P < 0.05, **P < 0.01, ***P < 0.001, ****P < 0.0001, and ns = no significance. Statistical analyses in (D) and (E) were performed using one-way ANOVA [file 12951_2026_4365_MOESM9_ESM.pdf]

**A**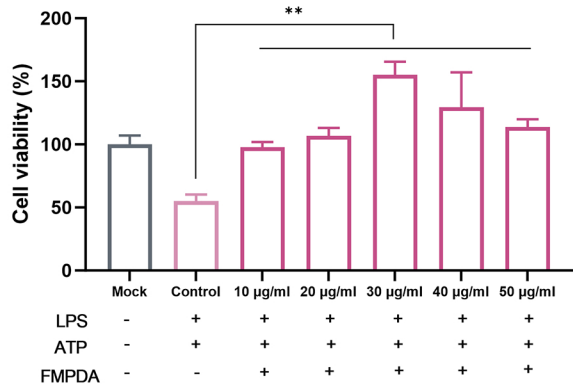**B**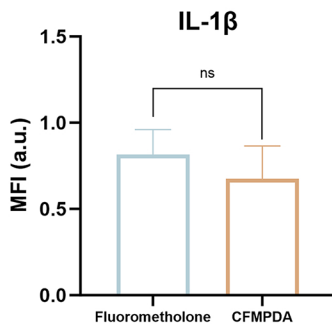**C**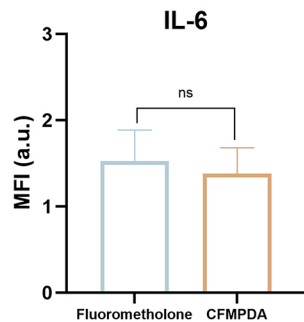**D**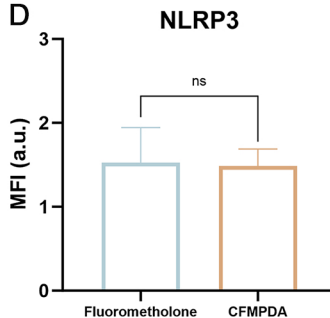**E**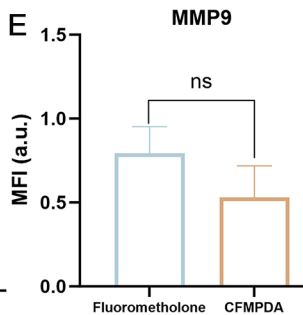**F**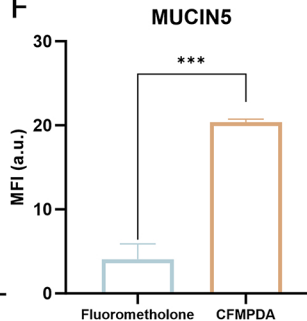**G**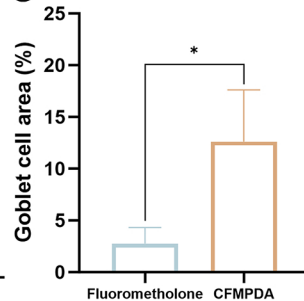

Supplement: Supplementary file 10 — Supplementary Material 10: Fig. S9 (A) CCK-8 assay demonstrating the optimal therapeutic concentration of FMPDA in LPS+ATP-induced HCECs. (B–E) MFI quantification of IL-1β, IL-6, NLRP3, and MMP-9 immunofluorescence signals in corneal sections. (F) MFI quantification of MUCIN5AC immunofluorescence signals in conjunctival sections. (G) Quantification of goblet cell area. All data are presented as mean ± SD (n = 4). Significance was set at *P < 0.05, **P < 0.01, ***P < 0.001, ****P < 0.0001, and ns = no significance. Statistical analyses were performed using one-way ANOVA (A) and two-tailed Student's t-test (B–G) [file 12951_2026_4365_MOESM10_ESM.pdf]

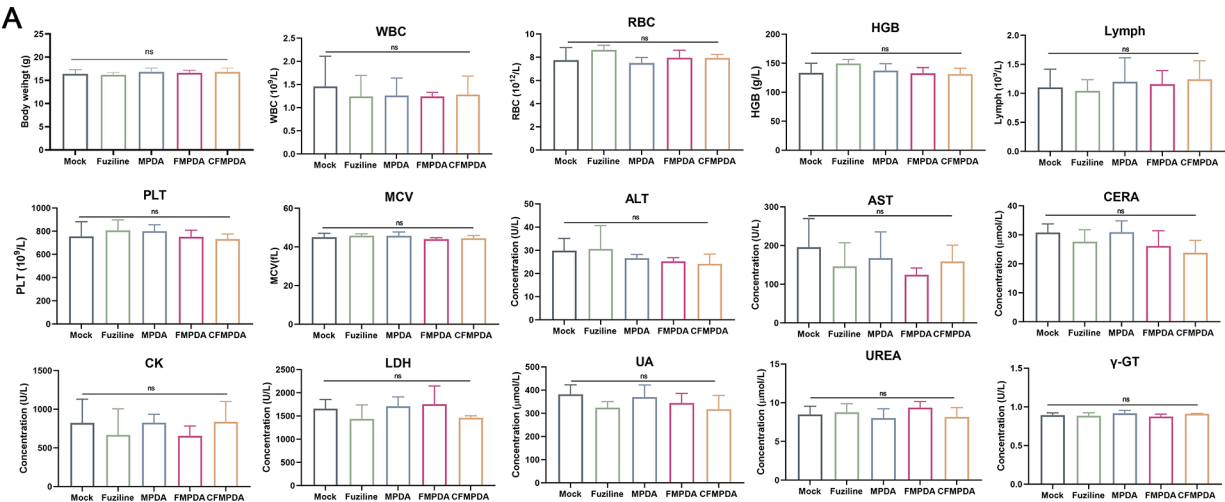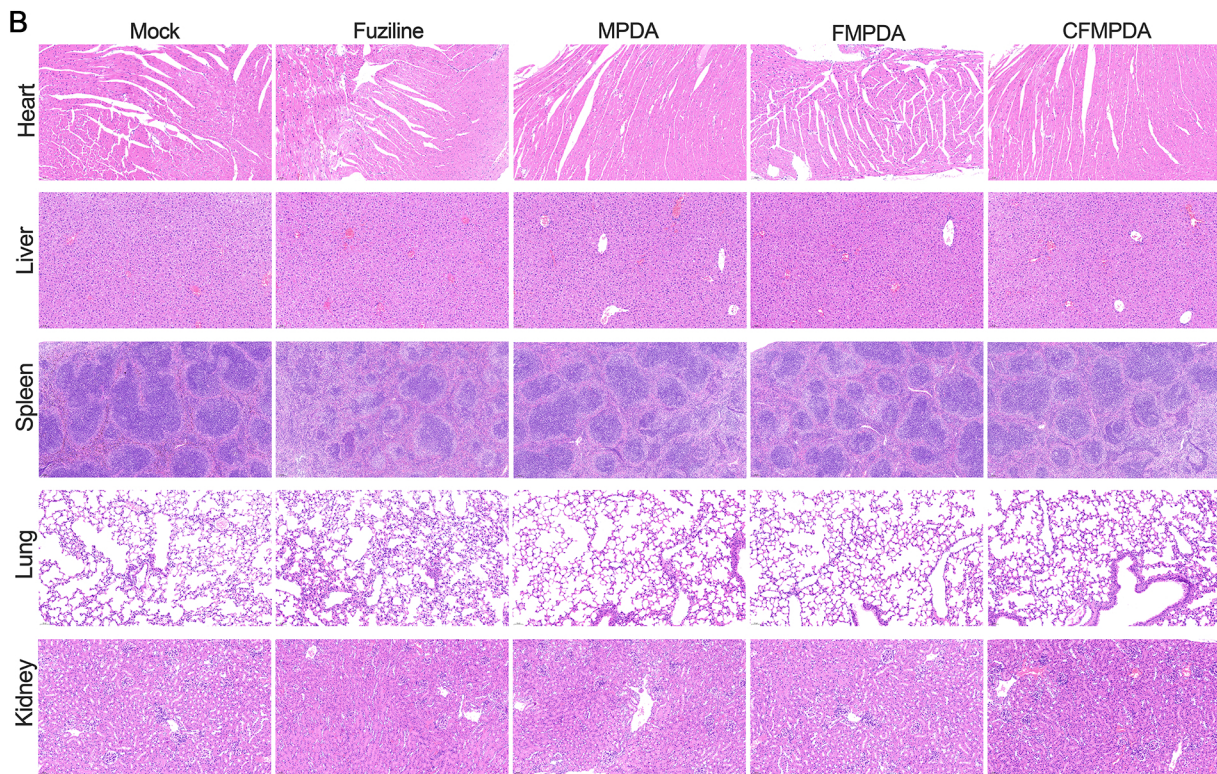

Supplement: Supplementary file 11 — Supplementary Material 11: Fig. S10 (A) Body weight and blood analysis of mice after various treatments for 14 days (n = 5). (B) H&E staining of histological sections of heart, liver, spleen, lung, and kidney samples collected from mice after various treatments for 14 days. All data are presented as mean ± SD (n = 5). Significance was set at *P < 0.05, **P < 0.01, ***P < 0.001, ****P < 0.0001, and ns = no significance. Statistical analysis in (A) was performed using one-way ANOVA [file 12951_2026_4365_MOESM11_ESM.pdf]
