# Supplementary material for: A sequentially targeted and pathology-responsive nanoplatform for synergistic treatment of dry eye disease via concurrent anti-inflammation and mitochondrial ROS scavenging
Source: J Nanobiotechnology. 2026 May 22;24:674. doi: 10.1186/s12951-026-04365-7 (PMC13383135; doi:10.1186/s12951-026-04365-7)
Supplement: Supplementary file 5 — Supplementary Material 5: Fig. S4 (A, B) Representative fluorescein sodium staining images and the corresponding staining score analysis. (C) MFI quantification of IL-1β and ROS immunofluorescence signals in corneal sections of the control and MPDA groups. (D) MFI quantification of IL-1β and ROS immunofluorescence signals in conjunctival sections of the control and MPDA groups. (E) TUNEL staining of corneal sections from the control and MPDA groups. (F) Quantification of TUNEL-positive cells in corneal sections. (G) TUNEL staining of conjunctival sections from the control and MPDA groups. (H) Quantification of TUNEL-positive cells in conjunctival sections. All data are presented as mean ± SD (n = 3). Significance was set at *P < 0.05, **P < 0.01, ***P < 0.001, and ****P < 0.0001. Statistical analyses in (B–D, F, H) were performed using a two-tailed Student's t-test [file 12951_2026_4365_MOESM5_ESM.pdf]

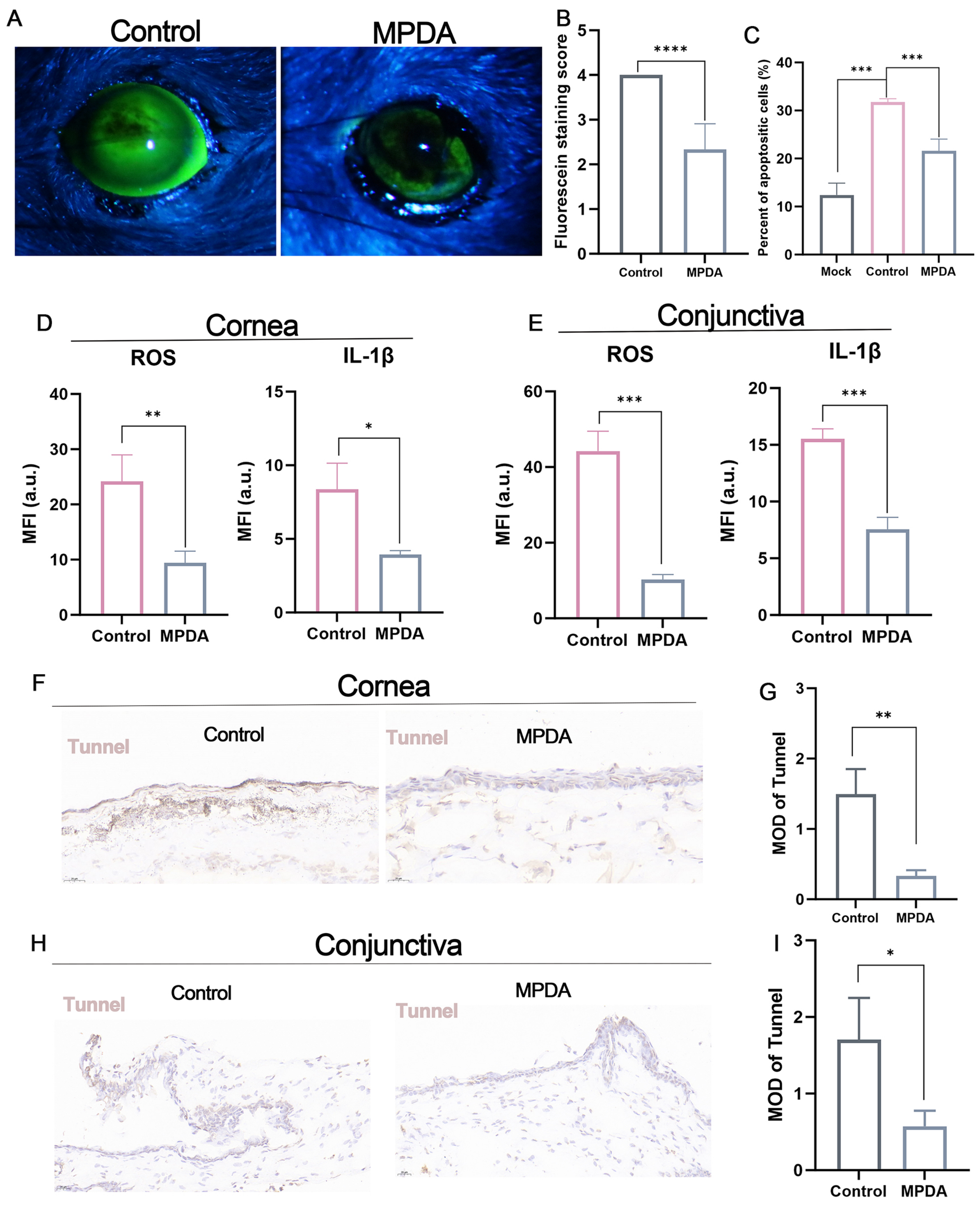

A
Control
MPDA
B
Fluorescein staining score
5
4
3
2
1
0
****
Control
MPDA
C
Percent of apoptositic cells (%)
40
30
20
10
0
***
***
Mock
Control
MPDA
D
Cornea
ROS
IL-1β
MFI (a.u.)
40
30
20
10
0
**
Control
MPDA
MFI (a.u.)
15
10
5
0
*
Control
MPDA
E
Conjunctiva
ROS
IL-1β
MFI (a.u.)
60
40
20
0
***
Control
MPDA
MFI (a.u.)
20
15
10
5
0
***
Control
MPDA
F
Cornea
Tunnel
Control
Tunnel
MPDA
G
MOD of Tunnel
3
2
1
0
**
Control
MPDA
H
Conjunctiva
Tunnel
Control
MPDA
Tunnel
I
MOD of Tunnel
3
2
1
0
*
Control
MPDA
