# Supplementary material for: A sequentially targeted and pathology-responsive nanoplatform for synergistic treatment of dry eye disease via concurrent anti-inflammation and mitochondrial ROS scavenging
Source: J Nanobiotechnology. 2026 May 22;24:674. doi: 10.1186/s12951-026-04365-7 (PMC13383135; doi:10.1186/s12951-026-04365-7)
Supplement: Supplementary file 2 — Supplementary Material 2 : Fig. S1 (A) Chord diagram showing GO enrichment analysis of the top 11 hub cytokines. (B) Concentrations of 64 cytokines in the supernatant of normal HCECs and LPS+ATP-induced HCECs. (C) STRING protein–protein interaction network of cytokines with a confidence score ≥1000. (D) Heatmap showing interactions among the top 14 hub cytokines. (E) Chord diagram showing GO enrichment analysis of the top 14 hub cytokines. [file 12951_2026_4365_MOESM2_ESM.pdf]

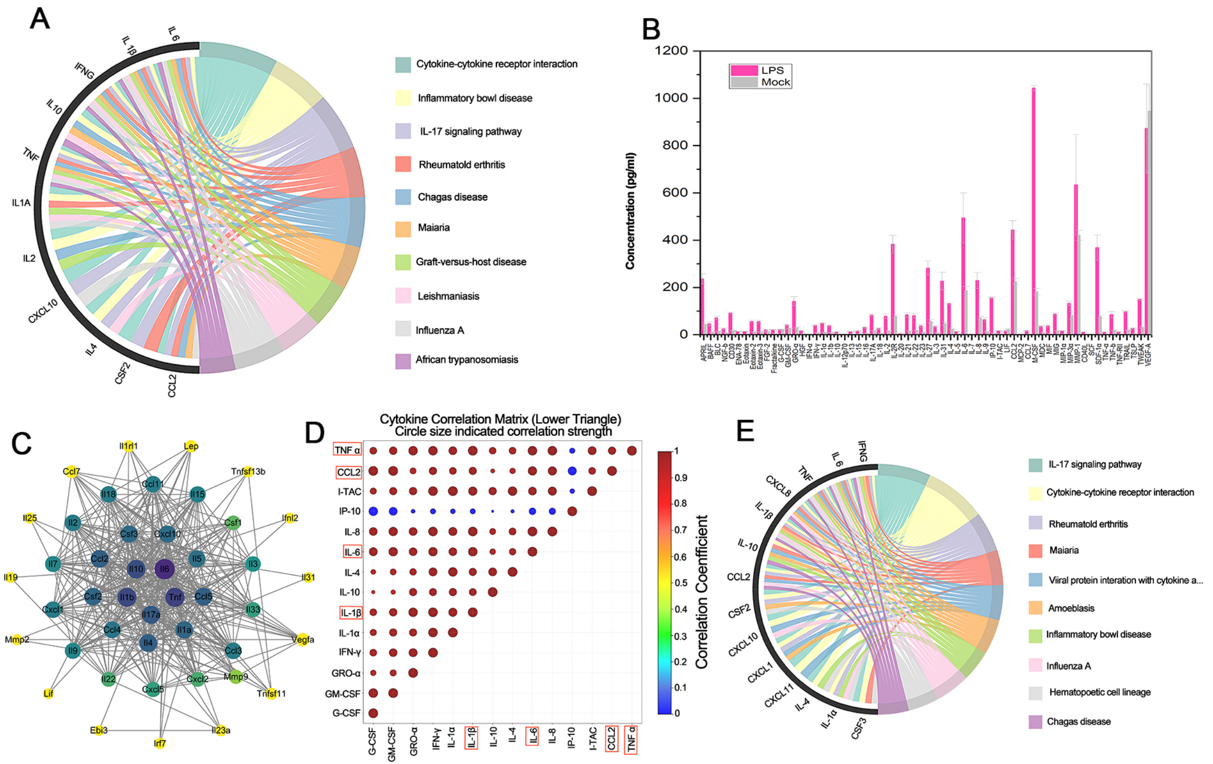
A
Cytokine-cytokine receptor interaction
Inflammatory bowl disease
IL-17 signaling pathway
Rheumatoid erthritis
Chagas disease
Maiaria
Graft-versus-host disease
Leishmaniasis
Influenza A
African trypanosomiasis
B
Concentration (pg/ml)
LPS
Mock
C
D
Cytokine Correlation Matrix (Lower Triangle)
Circle size indicated correlation strength
Correlation Coefficient
E
IL-17 signaling pathway
Cytokine-cytokine receptor interaction
Rheumatoid erthritis
Maiaria
Viiral protein interation with cytokine a...
Amoeblasis
Inflammatory bowl disease
Influenza A
Hematopoetic cell lineage
Chagas disease
